# Nanobody-Targeted Conditional Antimicrobial Therapeutics

**DOI:** 10.1021/acsnano.4c16007

**Published:** 2025-03-05

**Authors:** Chayanon Ngambenjawong, Henry Ko, Tahoura Samad, Novalia Pishesha, Hidde L. Ploegh, Sangeeta N. Bhatia

**Affiliations:** 1 Koch Institute for Integrative Cancer Research, Massachusetts Institute of Technology, Cambridge, Massachusetts 02139, United States; 2 Institute for Medical Engineering and Science, Massachusetts Institute of Technology, Cambridge, Massachusetts 02139, United States; 3 School of Biomolecular Science and Engineering, Vidyasirimedhi Institute of Science and Technology (VISTEC), Rayong 21210, Thailand; 4 Division of Immunology, Boston Children’s Hospital, Harvard Medical School, Boston, Massachusetts 02115, United States; 5 Program in Cellular and Molecular Medicine, Boston Children’s Hospital, Harvard Medical School, Boston, Massachusetts 02115, United States; 6 Howard Hughes Medical Institute, Cambridge, Massachusetts 02139, United States; 7 Department of Electrical Engineering and Computer Science, Massachusetts Institute of Technology, Cambridge, Massachusetts 02139, United States; 8 Department of Medicine, Brigham and Women’s Hospital and Harvard Medical School, Boston, Massachusetts 02115, United States; 9 Broad Institute of Massachusetts Institute of Technology and Harvard, Cambridge, Massachusetts 02139, United States

**Keywords:** conditional therapeutic, nanobody, conjugate, protease, Ly6G/C, ADAM10, bacterial infection

## Abstract

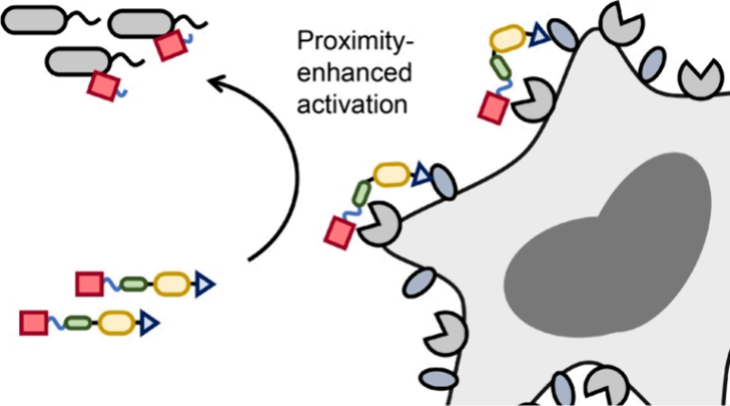

Conditional therapeutics that rely on disease microenvironment-specific triggers for activation are a promising strategy to improve therapeutic cargos. Among the investigated triggers, protease activity is used most often because of its dysregulation in several diseases. How to optimally fine-tune protease activation for different therapeutic cargos remains a challenge. Here, we designed nanobody-targeted conditional antimicrobial therapeutics to deliver a model therapeutic peptide and protein to the site of bacterial infection. We explored several parameters that influence proteolytic activation. We report the use of targeting nanobodies to enhance the activation of therapeutics that are otherwise activated inefficiently despite extensive optimization of the cleavable linker. Specifically, the pairing of Ly6G/C or ADAM10-targeting nanobodies with ADAM10-cleavable linkers improved activation via proximity-enabled reactivity. This study demonstrates a distinct role of active targeting in conditional therapeutic activation. More broadly, this optimization framework provides a guideline for the development of conditional therapeutics to treat various diseases in which protease activity is dysregulated.

## Introduction

Most investigational therapeutics fail in clinical trials because they lack efficacy or show unacceptable toxicity.^1−4^ Therapeutics with improved properties are identified through modification of their molecular properties and screening.^5,6^ Formulation of therapeutics with various drug delivery systems (e.g., encapsulation into or conjugation to nanocarriers or biomacromolecules) may also lead to improvement in efficacy.^7^ Activatable or conditional therapeutics are a promising therapeutic modality to enhance the efficacy or the safety profile of small-molecule drugs and biologics.^8−11^ Specific triggers (e.g., pH,^12,13^ hypoxia,^14^ redox,^15^ reactive oxygen species,^16^ and enzymes^8,9,17^) at the diseased sites are exploited to release or convert protherapeutics into their active forms. Protease aberration/involvement in the pathology of various diseases has made protease-activated therapeutics a major focus for the development of conditional therapeutics both preclinically and clinically.^8,9,18^

Notable examples of conditional therapeutics in advanced preclinical stages and clinical trials include Probody,^19^ precision-activated T-cell engager (XPAT),^20^ COBRA,^21^ and Indukine.^22^ These therapeutics encompass antibodies, T-cell engagers, and cytokines whose active binding sites are blocked by linking them to masking peptides, polypeptides, or proteins via protease-cleavable linkers. Being conditionally inactive, these therapeutics rely on passive targeting to accumulate at the diseased site(s) and engage disease-localized extracellular proteases.^8,9,18^ Selection of suitable protease-cleavable linkers ensures sufficient on-target activation at the site(s) of the disease, with minimal off-target activation. Most reports primarily selected cleavable linkers based on previous reports of disease biology or based on *in vitro* cleavage assays with biospecimens.^19−22^ Strategies for the selection and validation of the cleavable linkers in relevant *in vivo* models are still relatively limited, creating a knowledge gap that needs to be bridged to optimize this therapeutic modality. The incorporation of active targeting domains may improve conditional therapeutics, but how this influences accumulation and activation of conditional therapeutics at the site(s) of disease is underexplored. In addition, how different drug carrier compositions affect the activation of a conditional therapeutic is not known. The knowledge and assessment of how each component of a conditional therapeutic affects the *in vivo* activation and performance of the therapeutics will help inform the design parameters that can guide much-needed optimization of future therapeutics.

Here, we report the development and optimization of nanobody (VHH)-targeted conditional antimicrobial therapeutics for the conditional delivery of therapeutic peptide and protein cargos to the site of bacterial infection. We systemically investigated the effect of each domain component on the conditional activation of the therapeutics. In a mouse model of *Pseudomonas aeruginosa* infection of the lung, we found that Ly6G/C- or ADAM10-targeting VHHs could be used to enhance the conditional activation of the tethered therapeutic peptide/protein at the site of infection when paired with ADAM10-cleavable linkers. This enhanced activation was attributed to the closer proximity between VHH-targeted therapeutics and the target protease (ADAM10) rather than to the increased therapeutic accumulation in the infected organ. The study uncovers a distinct role of active targeting in conditional therapeutic activation. We provide a framework for the systematic optimization of targeted conditional therapeutics, applicable to diverse disease areas, especially those that involve the upregulation of ADAM10, such as infection^23−25^ and cancer.^26,27^

## Results and Discussion

### Design, Synthesis, and Mechanistic Concept of VHH-Targeted Conditional Antimicrobial Therapeutics

**Figure 1:**
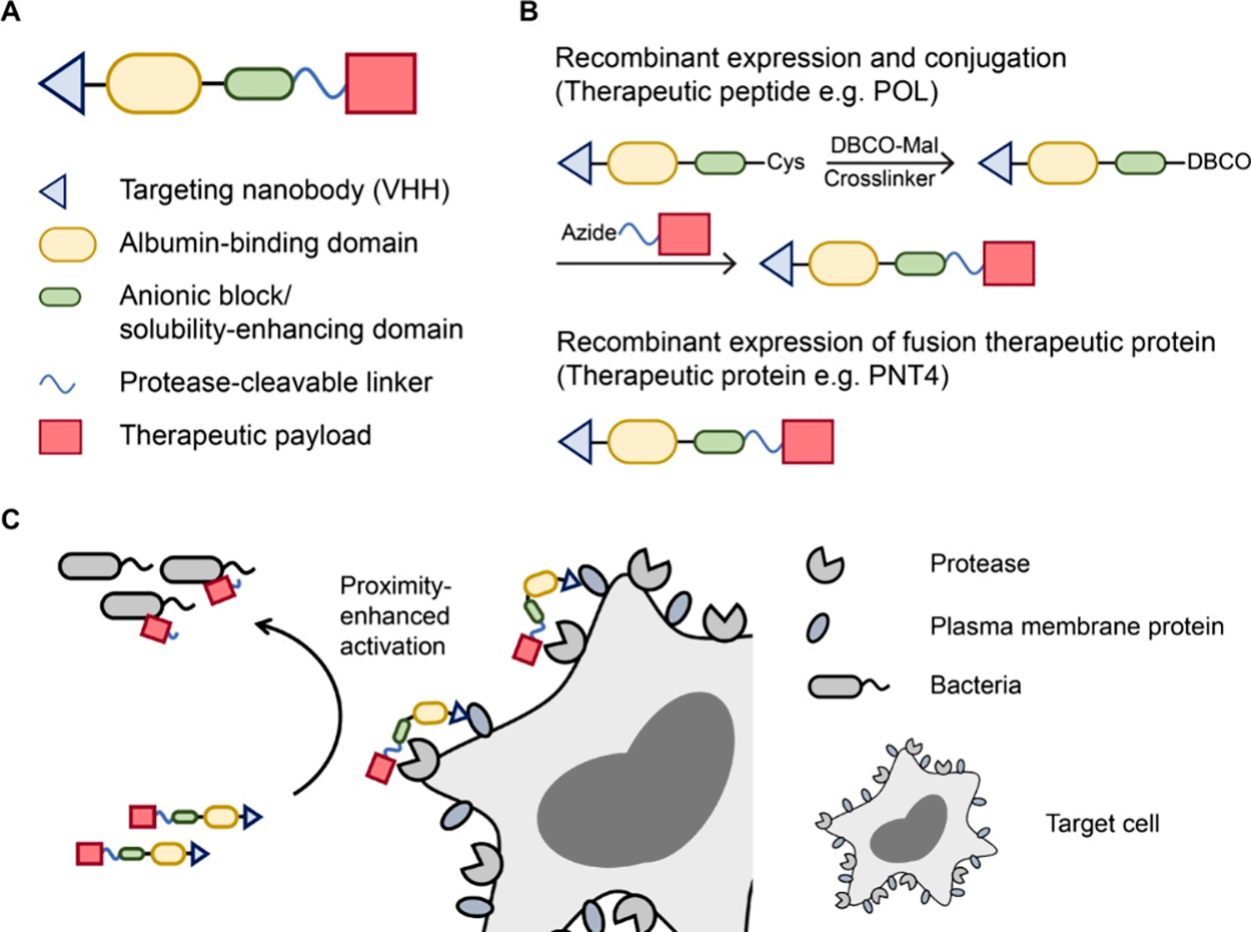
Design, synthesis, and mechanistic concept of VHH-targeted conditional antimicrobial therapeutics. (A) Design and (B) synthesis of VHH-targeted conditional antimicrobial therapeutics. (C) Proposed mechanism of enhanced conjugate activation via VHH targeting.

We previously reported the development of conditional antimicrobial peptide (AMP) therapeutics based on albumin-binding domain (ABD)-AMP conjugates. These conjugates lack a targeting moiety, passively accumulated at the site of bacterial lung infection, and were selectively activated by dysregulated proteases in the affected microenvironment.^28^ Given the clinical efficacy of tumor-targeting antibody-drug conjugates (ADCs) in oncology,^29−31^ antimicrobial therapeutics might similarly benefit from an active targeting domain. Here, we investigated the effect of VHH-mediated targeting on the biodistribution and efficacy of the conditional therapeutics using both therapeutic peptide and protein cargos. Our targeted conditional antimicrobial therapeutics are composed of (1) a VHH-based active targeting domain, (2) an ABD,^32^ (3) an anionic block/solubility-enhancing domain (EEG)_6_,^28^ (4) a protease-cleavable linker (Sx), and (5) the therapeutic payload (Figure 1A). We used two model cargos (LptD inhibitor POL7080/murepavadin (POL) as a therapeutic peptide and pyocin S2 N-terminal domain-T4 lysozyme (PNT4) as a therapeutic protein).

The conditional antimicrobial therapeutics were synthesized via (1) conjugation of the VHH-ABD carrier to the peptide cargo or (2) recombinant expression of the full fusion protein by combining the VHH-ABD carrier with the therapeutic protein cargo (Figure 1B). For the former, the VHH-(ABD)_2_-(EEG)_6_-Cys carrier domain was recombinantly expressed in *E. coli*, selectively functionalized with dibenzocyclooctyne-maleimide (DBCO-Mal) at the C-terminal Cys, and conjugated to an azide-functionalized cleavable linker-POL fusion peptide (Table S1) via a copper-free azide–alkyne cycloaddition. The fusion peptide was synthesized separately via a standard solid-phase peptide synthesis (SPPS).

We investigated VHHs whose binding targets (e.g., Ly6G/C, CD11b, ADAM10, and ADAM17) are enriched at the site of infection.^24,33,34^ As a model, we used *Pseudomonas aeruginosa* strain O1 (PAO1) to establish a pulmonary infection in mice. We hypothesized that active targeting via VHHs might enhance the interaction between infection-localized proteases and the cleavable linker by a proximity effect and thus increase the activation of the conditional antimicrobial therapeutics (Figure 1C).

### Active Targeting with Ly6G/C-Targeting VHH16 Enhances Conditional Antimicrobial Therapeutic Activation

**Figure 2:**
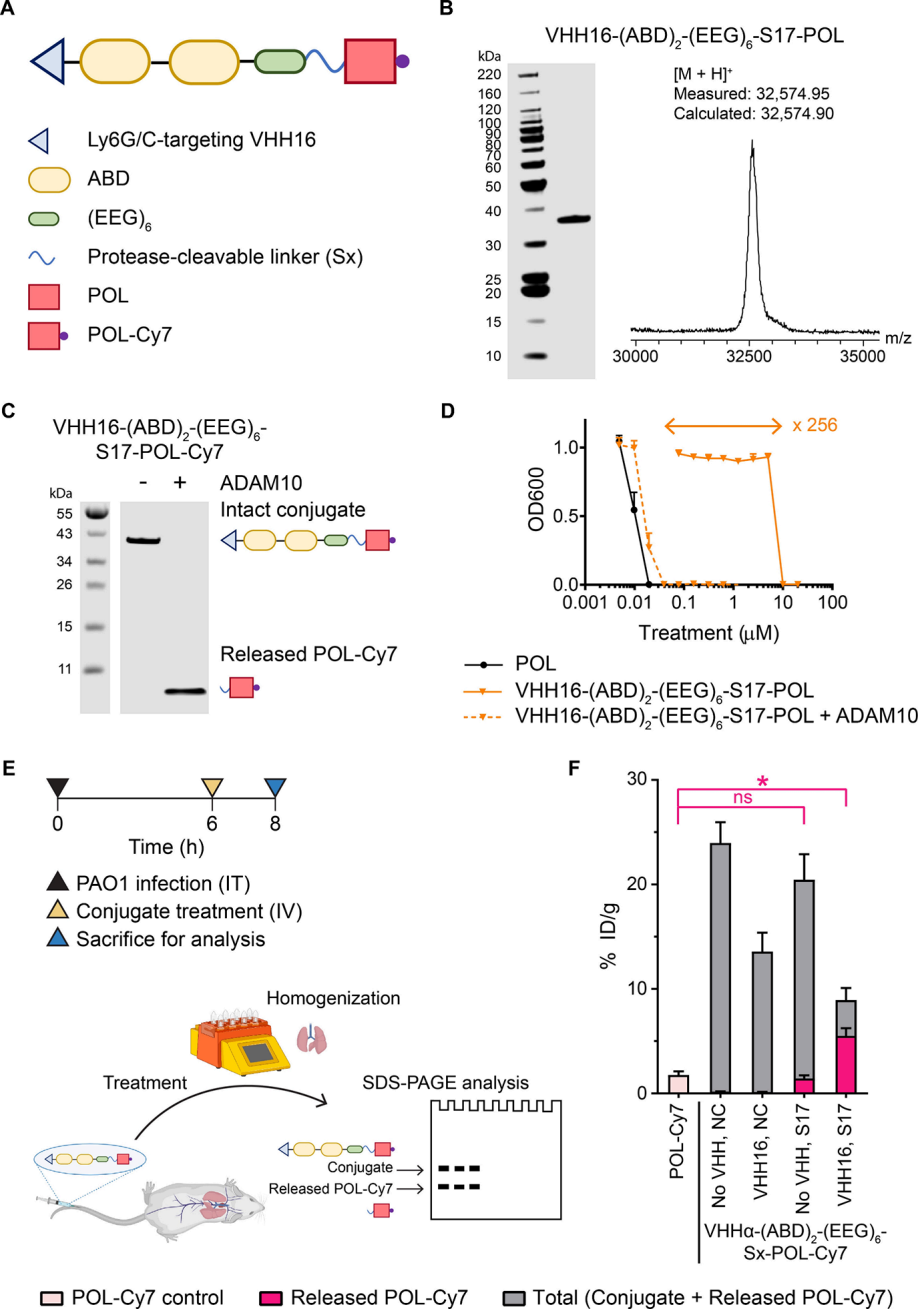
Inclusion of the Ly6G/C-specific VHH16 enhances the activation of the conditional antimicrobial therapeutic peptide. (A) Design of a conditional antimicrobial therapeutic for delivery of POL. (B) Characterization of the POL conjugate VHH16-(ABD)_2_-(EEG)_6_-S17-POL (left: SDS-PAGE; Coomassie blue staining, right: analysis by MALDI-ToF MS reported as mass-to-charge ratio *m*/*z*). (C) *In vitro* cleavage assay of VHH16-(ABD)_2_-(EEG)_6_-S17-POL-Cy7 by ADAM10 detected via Cy7 fluorescence using an Odyssey CLx imager. (D) *In vitro* evaluation of masking of antimicrobial activity by VHH16-(ABD)_2_-(EEG)_6_-S17-POL in a microdilution assay on PAO1. Bacterial viabilities were measured based on OD600 absorbance measurements normalized to the untreated control. (E) Experimental timeline and workflow for *in vivo* evaluation of biodistribution and activation of POL-Cy7 conjugates. (F) Quantification of total and activated fractions of the POL-Cy7 conjugates in PAO1-infected lungs presented as % injected dose (ID)/ gram (g). Panels D and F were plotted as mean ± standard deviation (SD) (*n* = 3). Panel F was analyzed with one-way ANOVA with Tukey *post hoc* tests. Selected comparisons between POL-Cy7 and released POL-Cy7 from the S17 conjugates were shown in pink. The asterisk (*) denotes statistical significance (*P* < 0.05). Panel E was partly created with BioRender.com.

Given the rapid influx of neutrophils at the site of infection,^33^ we first evaluated VHH16, a nanobody that recognizes Ly6G/C, present primarily on monocytes and neutrophils.^35,36^ The schematic of our POL-based targeted antimicrobial conjugate VHH16-(ABD)_2_-(EEG)_6_-S17-POL is shown in Figure 2A. We previously reported an *in vivo* screening pipeline of cleavable linkers in PAO1-infected lungs using ABD-AMP conjugates (ABD)_2_-(EEG)_6_-Sx-(D)Pex-Cy7, where (D)Pex-Cy7 served as a model fluorescently labeled linear AMP.^28^ In brief, (ABD)_2_-(EEG)_6_-Sx-(D)Pex-Cy7 conjugates with different cleavable linkers (Sx) were intravenously injected into PAO1-infected mice that were then euthanized after 2 h. The infected lungs were collected, homogenized, and analyzed for the presence of intact conjugate as well as released (D)Pex-Cy7 via SDS-PAGE, expressed as a fraction of the injected dose per gram. Subsequently, we searched published accounts for potential proteases that might cleave the lead linker from our initial study and nominated additional substrates that could be cleaved by those proteases for subsequent rounds of evaluation. In this study, we performed three additional rounds of *in vivo* cleavable linker screening (linkers S1–S17) and identified S17 as an improved substrate (Figure S1A–D). This substrate could be cleaved by ADAM10/17.^37^ While (ABD)_2_-(EEG)_6_-S17-(D)Pex-Cy7 was efficiently activated in the infected lungs (Figure S1D), the POL conjugate analog (ABD)_2_-(EEG)_6_-S17-POL-Cy7 was poorly activated, possibly due to the increased steric bulk of cyclic peptides (Figure S1E). The need to improve the activation of the POL conjugate suggested the inclusion of the VHH targeting domain. We first verified that fluorescently labeled VHH16 accumulated preferentially in PAO1-infected lungs in an infection-dependent, VHH-specific manner (Figure S2). VHH16-(ABD)_2_-(EEG)_6_-S17-POL was readily synthesized. Its identity was confirmed by SDS-PAGE and matrix-assisted laser desorption/ionization-time-of-flight (MALDI-ToF) mass spectrometry (MS) (Figure 2B). The fluorescent version was synthesized by using Cy7-labeled POL (Table S1) to enable fluorescent tracking of intact VHH16-(ABD)_2_-(EEG)_6_-S17-POL-Cy7 versus released POL-Cy7, as shown by ADAM10 cleavage (Figure 2C). We confirmed that the cleavage of VHH16-(ABD)_2_-(EEG)_6_-S17-POL by ADAM10 conferred conditional activation of the antibacterial activity of POL, showing a 256-fold difference in minimal inhibitory concentrations (MICs) between the intact and activated conjugates (Figure 2D).

To evaluate the biodistribution and activation of VHH16-targeted POL conjugates, non-neutropenic mice were infected intratracheally with PAO1 for 6 h before intravenous treatment with POL-Cy7 conjugates (Figure 2E). Mice were euthanized 2 h after conjugate treatment to harvest and homogenize the infected lungs for subsequent SDS-PAGE quantification of the amounts of the intact conjugate and released POL-Cy7. The noncleavable and non-VHH control conjugates were included to evaluate both the total accumulation and the effect of active targeting. Even though VHH16 itself preferentially accumulated in the PAO1-infected lungs compared with a control nanobody that binds an irrelevant target (Figure S2), VHH16-(ABD)_2_-(EEG)_6_-NC-POL-Cy7 with a noncleavable linker (NC) did not improve accumulation in the infected lungs compared to nontargeted (ABD)_2_-(EEG)_6_-NC-POL-Cy7 without VHH16 (Figure 2F and Figure S3). VHH16-(EEG)_6_-NC-POL-Cy7, without ABD, exhibited lower accumulation than both targeted and nontargeted ABD conjugates in the infected lungs, implying that albumin hitchhiking had a more predominant influence on conjugate accumulation in the lungs (Figure S3). Serendipitously, VHH16-(ABD)_2_-(EEG)_6_-S17-POL-Cy7 with a cleavable linker, S17, was found to be readily activated in the infected lungs, releasing ∼4-fold more POL-Cy7 compared to the amount released from the nontargeted (ABD)_2_-(EEG)_6_-S17-POL-Cy7 (Figure 2F). This enhanced activation of VHH16-(ABD)_2_-(EEG)_6_-S17-POL-Cy7 led to a 3-fold increase in released POL-Cy7 compared to that of the control (treatment with free POL-Cy7). We confirmed that ABD is required to achieve the high level of released POL-Cy7 in the infected lungs (Figure S4). Specifically, VHH16-(EEG)_6_-S17-POL-Cy7 without the ABD had an equivalent level of released POL-Cy7 as that of the free POL-Cy7 control in the infected lungs (Figure S4B). The conjugate was cleared via the kidneys (Figure S4D). The roles of active targeting of tumor/infection-targeting ADCs and immunocytokines include increased accumulation in the tumor/infected microenvironment and internalization by cancer/infected cells.^30,38,39^ In some cases, adding an active targeting domain did not improve the overall accumulation of drug carriers in a target organ, but it was found to help with target cell internalization.^40,41^ Some conditional therapeutics (e.g., Probody and XPAT) used in oncology have been designed with masking domains in the therapeutic constructs to inhibit active targeting and thereby avoid on-target, off-tumor accumulation and rely on passive accumulation before activation by tumor-associated proteases.^19,20^ Here, we report another unique role of active targeting in the context of protease-activated conditional therapeutics. We find that Ly6G/C-mediated targeting by VHH16 enhances the protease activation of its tethered therapeutic conjugate in the infected microenvironment.

### VHH16 Enhances the Activation of Conditional Antimicrobial Therapeutics in a VHH-Specific, ADAM10 Activity-Dependent Manner

**Figure 3:**
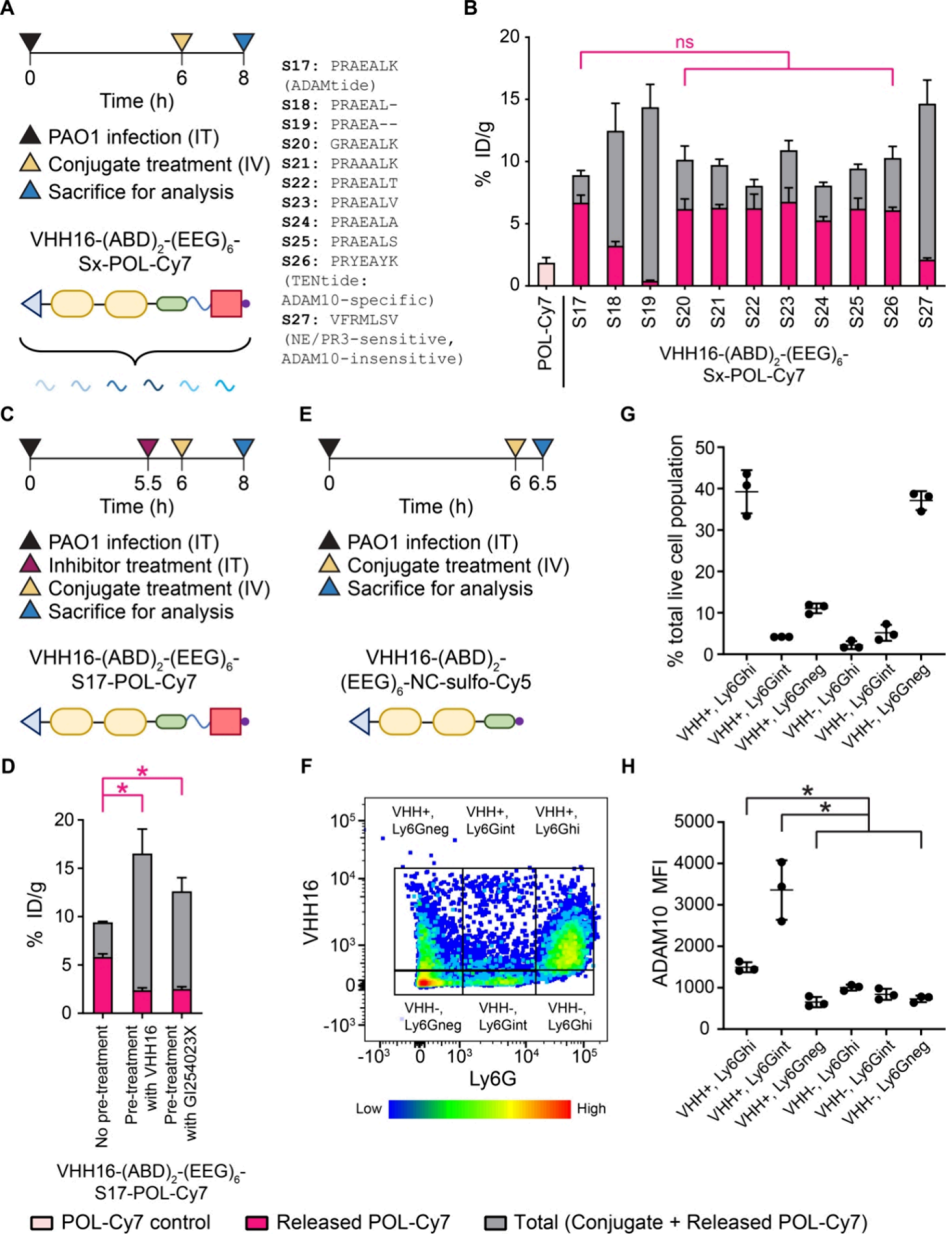
Enhanced activation of a VHH16-targeted conditional antimicrobial therapeutic requires interaction with the Ly6G/C target as well as proteolytic activity of ADAM10. (A) Experimental timeline for *in vivo* evaluation of the biodistribution and activation of VHH16-(ABD)_2_-(EEG)_6_-Sx-POL-Cy7 with different cleavable linkers (Sx). (B) Quantification of total and activated fractions of the POL-Cy7 conjugates in PAO1-infected lungs presented as % ID/g. (C) Experimental timeline for *in vivo* evaluation of biodistribution and activation of VHH16-(ABD)_2_-(EEG)_6_-S17-POL-Cy7 using intratracheal pretreatment with an excess either of VHH16-(ABD)_2_-(EEG)_6_ or of the ADAM10-selective inhibitor GI254023X. (D) Quantification of total and activated fractions of VHH16-(ABD)_2_-(EEG)_6_-S17-POL-Cy7 in PAO1-infected lungs presented as % ID/g. (E) Experimental timeline for analysis by flow cytometry of VHH16-(ABD)_2_-(EEG)_6_-NC-SulfoCy5 accumulation in different cell populations of PAO1-infected lungs. (F) A representative density plot of the lung cell populations based on *in vivo* accumulated VHH16 and *ex vivo* stained Ly6G using an anti-Ly6G monoclonal antibody. Gates were set based on the intensity of VHH16 (±) and Ly6G (hi/int/neg). (G) Quantification of each cell population presented as a percentage of the total live cell population. (H) Quantification of ADAM10 in each cell population based on *ex vivo* staining with an anti-ADAM10 monoclonal antibody, presented as median fluorescence intensity (MFI). Panels B, D, G, and H were plotted as mean ± SD (*n* = 3). Panels B, D, and H were analyzed with one-way ANOVA with Tukey *post hoc* tests. Selected comparisons between released POL-Cy7 from the S17 conjugate and the other conjugates were shown in pink. The asterisk (*) denotes statistical significance (*P* < 0.05).

To better understand the role of active targeting in the enhanced activation of conditional antimicrobial therapeutics, we focused on corroborating the identity of the proteases responsible for activation of the therapeutic conjugates. We expanded our screen for cleavable linkers by modification of substrate S17 by truncation or substitutions, as well as by inclusion of an ADAM10-specific linker S26 (TENtide)^37,42^ and a neutrophil elastase (NE)/proteinase 3 (PR3) cleavable linker S27 (Figure 3A). C-terminal truncations of the S17 linker (S18 and S19) progressively reduced the level of activation of VHH16-(ABD)_2_-(EEG)_6_-Sx-POL-Cy7 in PAO1-infected lungs. The deletion of the C-terminal Leu-Lys (S19) led to a near complete loss of conjugate activation (Figure 3B). This result corresponds to the *in vitro* cleavage assay of the conjugates with recombinant human ADAM10 (Figure S5) and aligns with the reported ADAM10/17 cleavage site between P1(Ala) and P1’(Leu).^37^ Matrix metalloproteinases (MMPs) have a substrate preference for Pro at the P3 position^43^ and could present an alternative cleavage site in the S17 substrate (PRA/EALK). P5(Pro-to-Gly) substitution (S20) showed that the Pro was not necessary for efficient *in vivo* activation, and hence, the cleavage was likely not primarily due to MMPs. Previous studies report the common appearance of Ala at the P2 position.^37,44^ P2(Glu-to-Ala) substitution (S21) was tested for possible improvement of *in vivo* activation, but no difference was observed compared to that of the S17 conjugate. Flexibility at the P2’ position allows the selection of cleavable linkers whose cleavage scars minimally affect the antimicrobial activity of the tethered therapeutics. We studied S22–S25, in which the original P2’ Lys was mutated to hydrophobic amino acids (Val/Ala) or hydroxyl amino acids (Thr/Ser). Each of these substitutions was well tolerated and did not affect the *in vivo* conjugate activation compared to the S17 conjugate. For the POL conjugates, the released POL with the P1’(Leu)-P2’(Thr) cleavage scar (e.g., cleavage from the linker S22) had an antimicrobial potency equivalent to that of the free POL, whereas the released POL with the P1’(Leu)-P2’(Lys) cleavage scar (from the initial S17 linker) had a 2-fold lower potency (Figure 2D and Figure S6). The conjugate with TENtide linker S26 was activated as efficiently as the one with the S17 linker, suggesting a contribution of ADAM10 in the infected lungs (Figure 3B). Even though NE is abundant in the infected microenvironment^33^ and the linker S17 can be cleaved by NE *in vitro* (Figure S7), the conjugate with a different NE-cleavable linker (S27),^45^ which is insensitive to ADAM10 cleavage (Figure S5 and S7), was not efficiently activated in the infected lungs. This points to a modest contribution, if any, of NE to the *in vivo* activation of VHH16-based conditional therapeutics (Figure 3B).

A follow-up biodistribution study was performed to investigate the effects of binding site saturation or inhibition of ADAM10 on the activation of VHH16-(ABD)_2_-(EEG)_6_-S17-POL-Cy7 *in vivo*. We administered intratracheally either VHH16-(ABD)_2_-(EEG)_6_ (10 equiv) or an ADAM10-selective inhibitor GI254023X, 30 min prior to intravenous treatment with VHH16-(ABD)_2_-(EEG)_6_-S17-POL-Cy7 (Figure 3C). Both lung-localized pretreatments reduced the activation of VHH16-(ABD)_2_-(EEG)_6_-S17-POL-Cy7, confirming a requirement for both VHH16-Ly6G/C-driven interactions as well as ADAM10 catalytic activity in the infected microenvironment (Figure 3D). ADAM10 exists in both transmembrane and soluble forms. We hypothesized that Ly6G/C and ADAM10 might be coexpressed on the same cell population such that the binding of VHH16 brings the conjugate closer to transmembrane ADAM10 and results in the proximity-enhanced activation of the conjugate. We therefore performed flow cytometry to evaluate the biodistribution of VHH16-(ABD)_2_-(EEG)_6_-NC-Sulfo-Cy5 in different lung cell populations. VHH16-(ABD)_2_-(EEG)_6_-NC-Sulfo-Cy5 was given intravenously to PAO1-infected mice followed by euthanasia to obtain lung single cell suspensions for flow cytometry with anti-Ly6G and anti-ADAM10 antibodies (Figure 3E). Gates set based on positivity for VHH16-(ABD)_2_-(EEG)_6_-NC-Sulfo-Cy5 and Ly6G separated lung cell suspension into six distinct populations, including VHH16+/VHH16– and Ly6Ghi (high)/int (intermediate)/neg (negative) (Figure 3F). VHH16 accumulated primarily in the Ly6G^hi^ population and, to a lesser extent, in the Ly6G^neg^ and Ly6G^int^ populations (Figure 3F,G). When analyzing these populations for the expression of ADAM10, the VHH16^+^Ly6G^int^ population showed the highest level of ADAM10 followed by the VHH16^+^Ly6G^hi^ population. The other cell populations express little, if any, ADAM10 (Figure 3H). Our flow cytometry study confirms the coexpression of Ly6G and ADAM10 on a subset of infected lung cell suspension that could therefore be targeted by VHH16, thus supporting our hypothesis of proximity-enhanced activation of such conjugates. While it is possible that the linkers investigated here are not exclusively specific to ADAM10/17, our mechanistic studies identified ADAM10 as the primary protease driving *in vivo* activation of our S17 conjugate. Indeed, expression of ADAM10 on monocytes/neutrophils has been reported and shown to be necessary for their migration into the alveolar space of lipopolysaccharide (LPS)-induced inflamed lungs.^23^

### Optimal Therapeutic Effect of VHH-Targeted Conditional Antimicrobial Therapeutic Requires the Optimization of Both VHH and the Cleavable Linker

**Figure 4:**
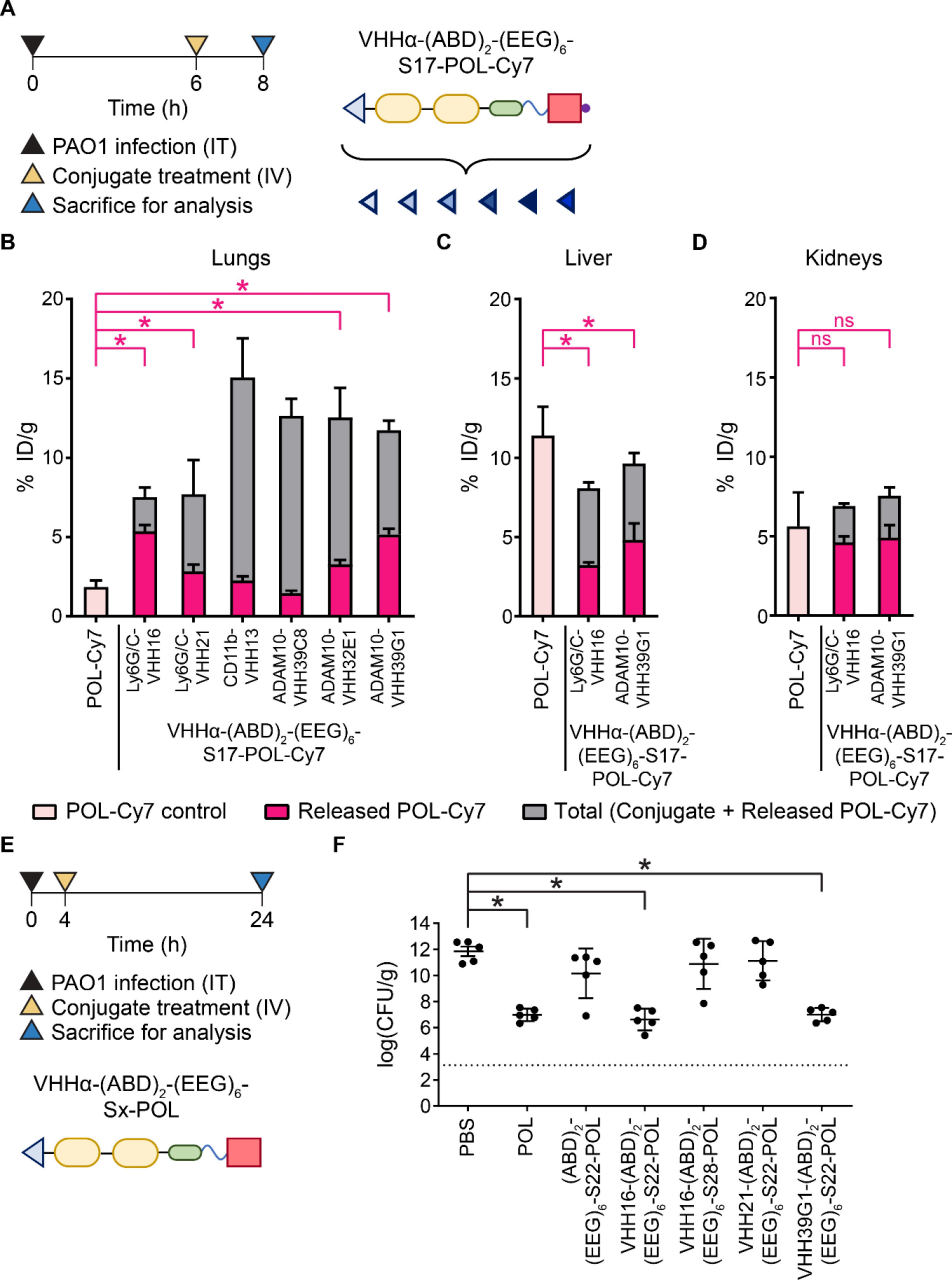
The therapeutic effect of VHH-targeted conditional therapeutics depends on the optimization of both VHH and the cleavable linker. (A) Experimental timeline for *in vivo* evaluation of the biodistribution and activation of VHHα-(ABD)_2_-(EEG)_6_-S17-POL-Cy7 with different targeting VHHs (VHHα). Quantification of the total and activated fractions of the POL-Cy7 conjugates in (B) PAO1-infected lungs, (C) liver, and (D) kidneys presented as % ID/g. (E) Experimental timeline for *in vivo* evaluation of the therapeutic efficacy of POL conjugates with different targeting VHHs and cleavable linkers. (F) Quantification of bacterial burden from the treated lungs presented as log of colony-forming unit (cfu)/g. Dotted lines denote the limit of detection. Panels B–D and F were plotted as mean ± SD (*n* = 3–4 for panels B–D; *n* = 5 for panel F). Panels B–D and F were analyzed with one-way ANOVA with Tukey *post hoc* tests. Selected comparisons between POL-Cy7 and released POL-Cy7 from the S17 conjugates were shown in pink. The asterisk (*) denotes statistical significance (*P* < 0.05). The evaluation of efficacy was confirmed in two independent studies with similar results.

Having observed that active targeting via VHH16 improved the activation of the conditional antimicrobial therapeutics, we expanded our investigation to include VHHs that recognize other potentially relevant targets (host: CD11b, ICAM-1, surfactant protein-A (SP-A), ADAM10, and ADAM17; pathogen: PcrV) (Figure 4A and Table S2). Another Ly6G/C-targeting VHH clone (VHH21)^35,36^ also improved the activation of the POL-based conjugate, albeit to a lower extent than the VHH16-based conjugate (Figure 4B). As seen for the Ly6G/C-targeting VHHs, ADAM10-targeting VHHs also enhanced conjugate activation in a clone-dependent manner. The enhanced activation conferred by the ADAM10 VHHs supports the validity of our hypothesis on the proximity-enhanced activity. In an *in vitro* cleavage assay with recombinant ADAM10, the ADAM10-targeted conjugates also showed increased activation kinetics, possibly due to the direct engagement between the ADAM10-targeting VHHs and the protease, bringing the conjugates closer for proximity-enhanced cleavage (Figure S8). The trend in cleavage efficiency, however, did not track with *in vivo* activation. Specifically, VHH39G1-(ABD)_2_-(EEG)_6_-S17-POL-Cy7 was the conjugate activated most efficiently *in vivo,* but it had the slowest kinetics of *in vitro* cleavage. This finding highlights the importance of *in vivo* validation to evaluate the extent of conjugate activation. VHHs for the other targets investigated (CD11b, ICAM-1, SP-A, and ADAM17) failed to improve conjugate activation (Figure 4B and Figure S9). The selected VHH clones may simply have been unable to display the conjugates in the appropriate geometry for proper proteolytic activation. Since the goal of conditional therapeutic development is selective activation at the site(s) of disease with minimal exposure in off-target organs, we evaluated the extent of conjugate activation of our lead conjugates in the liver and kidneys. For both VHH16-(ABD)_2_-(EEG)_6_-S17-POL-Cy7 and VHH39G1-(ABD)_2_-(EEG)_6_-S17-POL-Cy7, there is approximately 3-fold more release of POL-Cy7 than seen for the free POL-Cy7 treatment control in PAO1-infected lungs (Figure 4B). These levels were at least 2-fold lower in the liver (Figure 4C) and comparable in the kidneys (Figure 4D). POL, as an intravenous formulation, has failed a phase III clinical trial due to an increased incidence of acute kidney injury.^4^ There is currently no reported *in vivo* mouse model that recapitulates kidney toxicity induced by POL to guide the evaluation of toxicity for new formulations. Even though the current conjugates did not show signs of *in vivo* organ toxicity based on blood chemistry (Figure S10), future directions should focus on improvements in the design. This must be done to reduce the extent of off-target activation of conjugates in the kidneys. Investigation into coformulations with additives such as gelofusine or cilastatin might reduce kidney retention and thus yield further improvements.^46,47^

We next evaluated the therapeutic efficacy of the fully optimized conjugates comprising the optimized VHHs (VHH16 and VHH39G1) together with the optimized cleavable linker (S22). We included the conjugates with less optimized *in vivo* cleavage for comparison ((ABD)_2_-(EEG)_6_-S22-POL (nontargeted with the optimized linker), VHH21-(ABD)_2_-(EEG)_6_-S22-POL (suboptimal VHH clone with the optimized linker), and VHH16-(ABD)_2_-(EEG)_6_-S28-POL (optimal VHH clone with the NE-specific,^48^ non-ADAM10 cleavable linker)) (Figures 2F and 4B). Mice were infected intratracheally with PAO1 followed by treatment with conjugates 4 h after infection. At 24 h of infection, mice were euthanized to collect the infected lungs for enumeration of bacteria (Figure 4E). The conjugates that released the greatest levels of free drug (VHH16-(ABD)_2_-(EEG)_6_-S22-POL (Ly6G/C-targeting, ADAM10 cleavable linker) and VHH39G1-(ABD)_2_-(EEG)_6_-S22-POL (ADAM10-targeting, ADAM10 cleavable linker)) proved more effective than the conjugates with less optimal activation (Figure 4F) and performed comparably to free POL. Collectively, these data suggest that localizing activatable biologics to sites of infection and tuning the activation chemistry to the diseased microenvironment will improve therapeutic outcomes. These findings can be extended to activatable cytokines, T-cell engagers, checkpoint inhibitors, and ADCs across diseases from infection to oncology.

While it might be expected that the increased activation of our lead conjugates would show increased efficacy relative to free drug, we anticipate that the accurate assessment of the impact of increased local drug concentration over time will depend on the fidelity of animal models to recapitulate the time course of human disease progression. The disease progression in humans develops over a span of multiple days to weeks, and continuous antibiotic treatment is needed to maintain a high lung concentration of the therapeutic. Continuous treatment of POL has been shown to cause kidney toxicity in a clinical trial.^4^ Our formulation, which sustains a high concentration of free drug in the lungs for longer duration, may reduce the need for repeated drug dosing and thereby mitigate the toxicity issue. However, the mismatch between disease progression observed in humans (on the order of days) and in our current acute mouse infection model (on the order of hours), as well as the lack of POL toxicity in mice, precludes us from being able to accurately evaluate this multiday dosing optimization for its therapeutic efficacy and therapeutic index. Nonetheless, despite its limitations, this preclinical model offers the advantage of enabling quantitative comparisons of conjugate localization and activation as an important step in developing design principles for activatable conjugates. These studies can be extended in the future to models of infection that progress over days rather than hours. Future pursuits will also examine and optimize yet other parameters that may influence the efficacy of the conjugates. We show the importance of appropriate pairing between active targeting VHHs (Ly6G/C and ADAM10 binders) and cleavable linkers (ADAM10 substrates) to achieve enhanced conjugate activation. The lead VHHs in this study (Ly6G/C and ADAM10 binders) accumulated preferentially in the lungs of mice infected with PAO1 (Figures S2 and S11). Even so, further screens for VHHs that target a more infection-selective cell population (e.g., CD177 on activated neutrophils) or that interact with other dysregulated proteases in the diseased microenvironment (e.g., NE and PR3) may allow further improvement.^49−52^ Exploiting dysregulated proteases in the diseased microenvironment presents an additional avenue for conditional therapeutic development provided the optimal combination of the VHH target and cleavable linker can be identified.

### Active Targeting with Ly6G/C-Targeting VHH16 Enhances the Conditional Activation of a Model Therapeutic Protein (PNT4)

**Figure 5:**
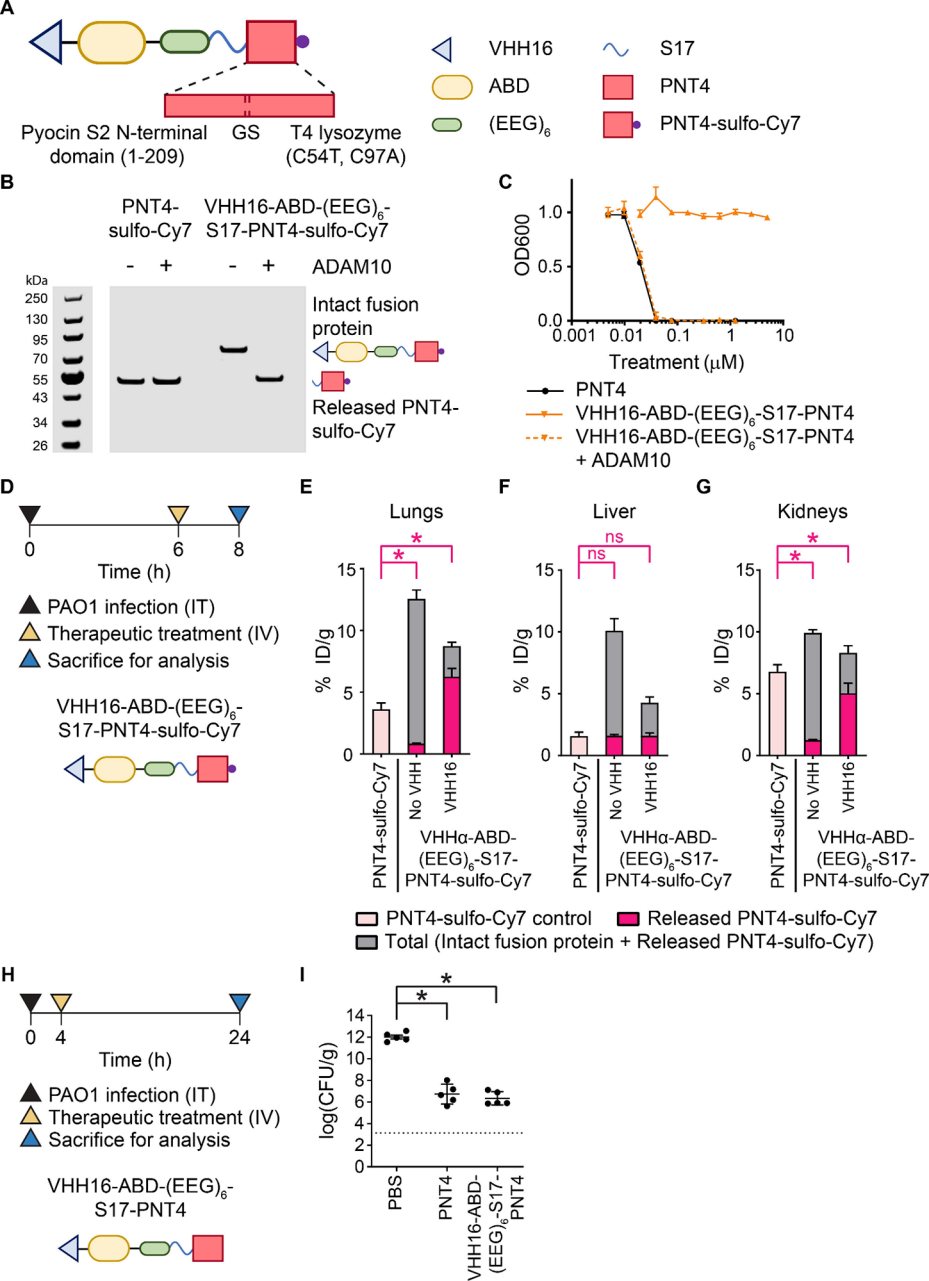
Demonstration of VHH16-enhanced activation of a conditional antimicrobial therapeutic protein. (A) Design of a conditional antimicrobial therapeutic for delivery of PNT4. (B) *In vitro* cleavage assay of VHH16-ABD-(EEG)_6_-S17-PNT4-sulfo-Cy7 by ADAM10 detected via sulfo-Cy7 fluorescence using an Odyssey CLx imager. (C) *In vitro* evaluation of antimicrobial activity masking of VHH16-ABD-(EEG)_6_-S17-PNT4 via a microdilution assay on PAO1. Bacteria viabilities were measured based on OD600 absorbance normalized to the untreated control. (D) Experimental timeline for *in vivo* evaluation of the biodistribution and activation of VHH16-ABD-(EEG)_6_-S17-PNT4-sulfo-Cy7. Quantification of the total and activated fractions of PNT4-sulfo-Cy7 in (E) PAO1-infected lungs, (F) liver, and (G) kidneys presented as % ID/g. (H) Experimental timeline for *in vivo* evaluation of the therapeutic efficacy of VHH16-ABD-(EEG)_6_-S17-PNT4. (I) Quantification of bacterial burden from the treated lungs presented as log(cfu/g). The dotted line denotes the limit of detection. Panels C, E–G, and I were plotted as mean ± SD (*n* = 3 for panels C and E–G; *n* = 5 for panel I). Panels E–G and I were analyzed with one-way ANOVA with Tukey *post hoc* tests. Selected comparisons between PNT4-sulfo-Cy7 and released PNT4-sulfo-Cy7 from the conditional therapeutics were shown in pink. The asterisk (*) denotes statistical significance (*P* < 0.05). The evaluation of efficacy was confirmed in two independent studies with similar results.

Following the optimization of the conditional antimicrobial therapeutic with a therapeutic peptide, POL, we investigated whether this design could be similarly adapted for the conditional delivery of a therapeutic protein. With their unique mechanism of action, engineered lysins are a potential alternative to antibiotics.^53,54^ Lysins hydrolyze peptidoglycan, readily killing Gram-positive bacteria, but are unable to penetrate the Gram-negative bacterial outer membrane and reach the periplasm where peptidoglycan is accessible for bactericidal action. Lysins fused to bacterial internalization domains enable their import into periplasm.^54−56^ We reasoned that by sterically blocking the internalization domain with the VHH-ABD fusion, it would be possible to formulate a conditional antimicrobial therapeutic. Here, we used the N-terminal domain of pyocin S2 (amino acids 1–209) to enable import of T4 lysozyme (catalytically active, disulfide-free mutant (C54T, C97A)^57^) into PAO1, which expresses the pyocin S2 receptor FvpAI^58^ (Figure 5A). We abbreviated this N-terminal pyocin S2 domain-T4 lysozyme fusion therapeutic protein as PNT4. The conditional therapeutic VHH16-ABD-(EEG)_6_-S17-PNT4 was readily expressed as a fusion protein in *E. coli* (Figure S12). The C-terminal sulfo-Cy7-labeled version was used to confirm conditional activation via cleavage by ADAM10 (Figure 5B), which corresponds to masking of conditional bactericidal activity (Figure 5C).

A biodistribution study in PAO1-infected mice was performed to compare VHH16-ABD-(EEG)_6_-S17-PNT4-sulfo-Cy7, ABD-(EEG)_6_-S17-PNT4-sulfo-Cy7, and PNT4-sulfo-Cy7 (Figure 5D). As observed for the POL conjugates, the nontargeted ABD-(EEG)_6_-S17-PNT4-sulfo-Cy7 was poorly activated in the infected lungs, whereas the addition of VHH16 (VHH16-ABD-(EEG)_6_-S17-PNT4-sulfo-Cy7) led to a 7.5-fold increase in activation (Figure 5E). VHH16-ABD-(EEG)_6_-S17-PNT4-sulfo-Cy7 released 1.7-fold more active protein in the infected lungs than did treatment with free PNT4-sulfo-Cy7. In the off-target organs, the active fractions were comparable in the liver (Figure 5F) and 1.4-fold lower for VHH16-ABD-(EEG)_6_-S17-PNT4-sulfo-Cy7 in the kidneys compared to those of the free PNT4-sulfo-Cy7 group (Figure 5G). Evaluation of the *in vivo* efficacy showed comparable potency for VHH16-ABD-(EEG)_6_-S17-PNT4 and free PNT4 (Figures 5H,I). It is possible that the fold difference in the active protein fractions is not sufficient to boost therapeutic efficacy in this infection model. Further optimization should be considered similar to what was described for the POL conjugates. PNT4 was well-tolerated in mice due to its unique specificity for the hydrolysis of peptidoglycan (Figure S13). The design of conditional antimicrobial therapeutics and the parameters for optimization could be applied to other therapeutic peptides or proteins where enhanced delivery is beneficial or where off-target toxicity might be a concern.

## Conclusions

We report the development of nanobody-targeted conditional antimicrobial therapeutics for the enhanced delivery of therapeutic antimicrobial peptide (POL) and protein (PNT4) to the site of infection. The pairing of VHHs that target Ly6G/C or ADAM10 with ADAM10-cleavable linkers was responsible for the improved activation. Analysis by flow cytometry of infected lung cell populations that bound Ly6G/C-specific VHH16 showed a subpopulation that coexpressed both Ly6G and ADAM10. This colocalization supports the postulated mechanism of improved activation via proximity-enhanced reactivity. The selection of Ly6G/C or ADAM10-specific VHHs as targeting moieties and the optimization of ADAM10 cleavable linkers were essential to improve the therapeutic efficacy of these conditional antimicrobial therapeutics. Other targeting domains (e.g., CD177/Ly6G/NE/PR3/MMP8 binders), cleavable linkers (e.g., NE/PR3/MMP8 substrates), and combinations merit consideration to further improve specificity for the infected microenvironment or to improve delivery of the active therapeutic payloads. Our platform and optimization framework are applicable to the development of conditional therapeutics for other disease areas with dysregulated proteases, including cancer, autoimmune diseases, and fibrosis.

## Materials and Methods

### Molecular Cloning

gBlocks gene fragments encoding fusion proteins flanked with *Nco*I and *Xho*I restriction sites were ordered from Integrated DNA Technologies (IA, U.S.A.). The gene fragments were cloned into a Novogen pET28a(+) plasmid vector at the *Nco*I and *Xho*I restriction sites via restriction enzyme digestion and ligation and transformed into NEB 5-alpha competent *E. coli*. Colonies containing correct sequences of the gene inserts were identified by Sanger sequencing via Quintara Biosciences (MA, U.S.A.) and grown overnight for miniprep extraction of plasmid DNA. The extracted plasmids were transformed into BL21(DE3) competent *E. coli* for nondisulfide bond-containing proteins or SHuffle T7 Express competent *E. coli* for disulfide bond-containing proteins (e.g., nanobody-containing fusion proteins).

### Recombinant Expression of Protein Therapeutics

Overnight primary cultures of BL21(DE3) or SHuffle T7 Express *E. coli* encoding the proteins of interest were expanded into 500 mL of secondary culture in Luria–Bertani (LB) broth (supplemented with 50 μg/mL kanamycin) and incubated in an incubator shaker at 220 rpm and 37 °C for 4–6 h until OD600 reached 0.6–0.8. The BL21(DE3) *E. coli* secondary culture was induced with 1 mM isopropyl β-d-1-thiogalactopyranoside (IPTG) for 2 h at 37 °C, while the SHuffle T7 Express *E. coli* secondary culture was induced with 0.4 mM IPTG for 24 h at 25 °C. At the end of the IPTG induction, bacteria were spun down at 4500 rpm for 15 min, frozen, and stored in a −80 °C freezer. For purification, the bacteria pellet was thawed on a 37 °C water bath, resuspended in a B-PER complete bacterial protein extraction reagent (15 mL), and incubated on a shaker for 15 min at room temperature (RT). The lysed bacteria suspension was spun down at 11,000 rpm for 20 min, and the supernatant was incubated in a Qiagen Ni-NTA agarose resin (1 mL) for 1 h at 4 °C to capture the His-tagged protein product. The resin suspension was poured into a fritted column, washed with 5 mL of wash buffer A (50 mM Tris, 500 mM NaCl, and 2% Triton X-114) and 5 mL of wash buffer B (50 mM Tris and 500 mM NaCl), and eluted with 1.5 mL of an elution buffer (50 mM Tris, 500 mM NaCl, 500 mM imidazole, and 10% glycerol). The eluted product was confirmed by SDS-PAGE using the NuPAGE 4 to 12% Bis–Tris mini protein gel stained with the Bio-Safe Coomassie stain.

### Site-Specific Conjugation of the Therapeutic Peptide or Fluorescent Dye

The selective reduction of the C-terminal cysteine of VHH-containing fusion protein was adapted from a previously described protocol.^59^ In brief, the protein solution (5–10 mg in 1.5 mL of elution buffer) was supplemented with EDTA (1 mM final concentration) and incubated in the Pierce immobilized TCEP disulfide reducing gel (0.25 mL, prewashed three times with 1 mL of PBS) with gentle rotation at RT for 24 h. Following the incubation, the gel was pelleted at 5000 rpm for 5 min. The supernatant was centrifuge-filtered to exchange the buffer into PBS (1 mM EDTA, pH 6.5) using 10 kDa Amicon centrifugal filter units (two filter units per 5 mg protein, 14,000 rpm spin for 2 min, four times). The protein solution was diluted in the same buffer to ∼5 mg/mL and reacted with DBCO-Mal (3 equiv) or fluorescent dye-maleimide (1.5 equiv) at RT for 4 h. The DBCO-functionalized or dye-labeled protein was purified using a Cytiva disposable PD-10 desalting column to remove unreacted DBCO-Mal/dye-maleimide and exchange the buffer for PBS (pH 7.4). Therapeutic peptides and their Cy7-labeled analogs (Table S1 and Figure S14) were synthesized by CPC Scientific (CA, U.S.A.) via standard Fmoc-based SPPS. For therapeutic peptide conjugation, DBCO-functionalized protein (10 mg) was first immobilized onto Qiagen Ni-NTA agarose (0.5 mL, prewashed thrice with 1 mL of PBS) at RT for 15 min. Azido-functionalized therapeutic peptide (2 equiv) was then added to the agarose suspension and incubated with gentle rotation at RT for 24 h. The agarose suspension was loaded into a fritted column, washed with the wash buffer B (10 mL) to remove unreacted peptide, and eluted with the elution buffer (1 mL). The conjugated product was buffer-exchanged into PBS (pH 7.4) using a PD-10 desalting column, and its identity and purity were confirmed by SDS-PAGE. The lead candidates were further characterized by MALDI-ToF MS.

### Protease Cleavage Assay

Dye-labeled antimicrobial therapeutics (VHHα-(ABD)_2_-(EEG)_6_-Sx-POL-Cy7 or VHH16-ABD-(EEG)_6_-S17-PNT4-sulfo-Cy7) were incubated with recombinant human ADAM10 (R&D Systems, MN, U.S.A.) at 10 μM and 250 nM final concentrations, respectively. Following the incubation at RT for 24 h, an aliquot (5 μL) was collected and diluted into PBS (1× Halt protease inhibitor cocktail, 1× EDTA) (15 μL) to stop the protease activity. SDS-PAGE was performed using the NuPAGE 4 to 12% Bis–Tris mini protein gel to separate cleaved and intact therapeutics and detect via Cy7/sulfo-Cy7 fluorescence using an Odyssey CLx imager at the 800 nm channel (LI-COR Biosciences, NE, U.S.A.).

### Microdilution Assay

*P. aeruginosa* strain PAO1 was a generous gift from the Ribbeck Lab at the Massachusetts Institute of Technology. A secondary culture of PAO1 was grown in a Mueller–Hinton broth (MHB) in an incubator shaker at 37 °C until the OD600 reached approximately 0.6. The bacteria culture was washed once in MHB, resuspended in MHB (1 mM human serum albumin (HSA)) to 10^6^ cfu/mL density, and plated on 96-well plates (50 μL/well). Twofold serial dilutions of antimicrobial therapeutic protein solutions (with or without preincubation in human ADAM10 (250 nM) for 8 h) were prepared in MHB and transferred to the bacteria-plated wells to the final volume of 100 μL (5 × 10^5^ cfu per well). The plates were incubated at 37 °C for 16 h before measurement of OD600 using an Infinite 200 PRO plate reader (Tecan, Switzerland) to determine bacteria viability.

### Mouse Model of Bacterial Lung Infection

All animal studies were approved by the Massachusetts Institute of Technology’s Committee on Animal Care (MIT CAC protocol 2203000310). For a neutropenic lung infection model, CD-1 mice (11–12 weeks old) were intraperitoneally administered cyclophosphamide at 4 and 1 days (150 and 100 mg/kg, respectively) prior to infection. On the infection day, a secondary culture of PAO1 (OD600 0.6–0.8) was washed twice in PBS, resuspended in PBS, and intratracheally administered to mice (2 × 10^5^ cfu in 50 μL PBS) using a 22G blunt-end catheter (EXCEL International). For a non-neutropenic lung infection model, the cyclophosphamide treatment was omitted, and the PAO1 suspension was intratracheally administered at the dose of 7.5 × 10^6^ cfu in 50 μL of PBS. All animal studies were performed in the non-neutropenic lung infection model unless specified as the neutropenic model.

### Biodistribution Study

After 6 h of bacterial lung infection, the infected mice were intravenously administered dye-labeled antimicrobial therapeutics (15 nmol) and euthanized 2 h later to collect the infected lungs, liver, and kidneys. The organs were transferred to gentleMACS M tubes and homogenized on a gentleMACS tissue dissociator using PBS (1× Halt protease inhibitor cocktail) as a medium. The homogenates were pelleted at 14,000 rpm for 30 min, and the supernatants were used for analysis. SDS-PAGE was performed using the NuPAGE 4 to 12% Bis–Tris mini protein gel to separate cleaved and intact therapeutics and detect via Cy7/sulfo-Cy7 fluorescence using an Odyssey CLx imager at the 800 nm channel. A representative gel data set is shown in Figure S15. Quantification (% ID/g) was determined using a standard curve from the stock solution of the dye-labeled therapeutics. For the biodistribution study with VHH16 competition or ADAM10 protease inhibitor, either VHH16-(ABD)_2_-(EEG)_6_ (10 equiv in 50 μL PBS) or the ADAM10-selective inhibitor GI254023X (MedChemExpress, NJ, U.S.A.) (5 mg/kg dose in 50 μL 0.9% NaCl (10% DMSO, 20% sulfobutylether-β-cyclodextrin)) was administered intratracheally at 5.5 h postinfection followed by intravenous treatment with VHH16-(ABD)_2_-(EEG)_6_-S17-POL-Cy7 (10 nmol) at 6 h postinfection.

### Flow Cytometry Analysis of VHH Biodistribution in Lungs

After 6 h of bacterial lung infection, the infected mice were intravenously administered VHH16-(ABD)_2_-(EEG)_6_-NC-sulfo-Cy5 without a cleavable linker (10 nmol) and euthanized 30 min later to collect the infected lungs. Single cell suspensions of the infected lungs were prepared as previously described.^60^ Briefly, the infected lungs were transferred to gentleMACS C tubes and homogenized on a gentleMACS tissue dissociator (“m_lung_01″ program) using the HEPES buffer (2 mg/mL collagenase D (Roche) and 80 U/mL DNase I (Roche)) as a medium. The homogenates were incubated in an incubator shaker at 37 °C for 30 min and further homogenized using the “m_lung_02″ program. Single cell suspensions were collected by filtering the homogenates through 70 μm mesh cell strainers. The single cell suspensions were centrifuged at 300*g* for 10 min and resuspended in PBS (3 mL). Aliquots of the single cell suspensions (5 × 10^6^ cells per aliquot) were first stained with the Zombie Aqua fixable viability kit (1:500 dilution in PBS) and Fc-blocked with the TruStain FcX (antimouse CD16/32) antibody (BioLegend, CA, U.S.A.; 1:20 dilution in PBS (1% BSA)) before further staining with the Alexa Fluor 488 antimouse Ly6G antibody (Clone 1A8, BioLegend, CA, U.S.A.; 1:100 dilution in PBS (1% BSA)) and DyLight 550 antimouse ADAM10 antibody (Clone RM0146-7H12, Novus Biologicals, CO, U.S.A.; 1:100 dilution in PBS (1% BSA)). The stained cell suspensions were fixed in 4% paraformaldehyde and resuspended in PBS (1% BSA) before flow cytometry analysis using a FACSymphony A3 cell analyzer (BD Biosciences, NJ, U.S.A.).

### *InVivo* Efficacy Evaluation

After 4 h of bacterial lung infection, the infected mice were intravenously administered antimicrobial therapeutics (2.5 mg/kg POL equiv for POL and POL conjugates and 0.25 mg/kg PNT4 equiv for PNT4 and VHH16-ABD-(EEG)_6_-PNT4). At 24 h postinfection, the mice were euthanized. The infected lungs were collected and homogenized on a gentleMACS tissue dissociator using gentleMACS M tubes with PBS as a medium. The homogenates were 10-fold serially diluted in PBS and plated on LB agar plates to determine cfu/g.

### *In Vivo* Toxicity Evaluation

Healthy CD-1 mice were intravenously administered antimicrobial therapeutics (5 mg/kg POL equiv per dose for POL and POL conjugates (two doses at 0 and 6 h time points) and 5 mg/kg PNT4 equiv for PNT4 and VHH16-ABD-(EEG)_6_-PNT4) and monitored for weight change and any signs of distress. At 24 h post-treatment, the mice were euthanized via cardiac puncture to draw blood for serum separation using Microtainer serum separator tubes. The serum samples were submitted to the MIT Division of Comparative Medicine Diagnostic Laboratory for serum chemistry analysis.

### Statistical Analysis and Schematic Representation

Statistical analysis was performed with the GraphPad Prism software. Data were plotted as mean ± SD. Comparisons among different treatment groups were based on one-way ANOVA with Tukey *post hoc* tests. A *P* value < 0.05 was considered statistical significance. A part of schematics in this publication was created with BioRender.com.

## Supporting Information Available

Optimization of the cleavable linker improves conditional therapeutic activation (Figure S1); VHH16 accumulated in PAO1-infected lungs in an infection-dependent, VHH-specific manner (Figure S2); VHH16 did not increase the accumulation of ABD conjugates in PAO1-infected lungs (Figure S3); ABD is required to increase the amount of released POL-Cy7 of VHH16-targeted conjugate in PAO1-infected lungs (Figure S4); the *in vitro* ADAM10 cleavage assay identified tolerable mutations of the S17 linker (Figure S5); activated VHH16-(ABD)_2_-(EEG)_6_-S22-POL has an equivalent antimicrobial potency as free POL (Figure S6); S17 and S27 POL-Cy7 conjugates can be activated to neutrophil elastase (Figure S7); ADAM10-targeting VHHs enhance the *in vitro* conjugate activation by ADAM10 (Figure S8); expanded screening of VHHs with relevant targets for enhanced conjugate activation (Figure S9); VHH-targeted POL conjugates exhibit good safety profiles (Figure S10); VHH39G1 accumulated in PAO1-infected lungs in an infection-dependent, VHH-specific manner (Figure S11); recombinant VHH16-ABD-(EEG)_6_-S17-PNT4 was readily expressed (Figure S12); VHH-targeted conditional PNT4 exhibits a good safety profile (Figure S13); aAn example of POL peptide for conjugation (Figure S14); a representative SDS-PAGE image of lung homogenates from the biodistribution study (Figure S15); list of therapeutic peptides and sequences (Table S1); and list of VHH clones (Table S2) (PDF)

## Notes

A previous version of this manuscript has been deposited as a preprint on bioRxiv. Chayanon Ngambenjawong; Henry Ko; Novalia Pishesha; Hidde L. Ploegh; Sangeeta N. Bhatia. Nanobody-targeted conditional antimicrobial therapeutics. 2024. bioRxiv. doi: 10.1101/2024.02.20.580917 (February 22, 2024).
