# Supplementary material for: Nanobody-Targeted Conditional Antimicrobial Therapeutics
Source: ACS Nano. 2025 Mar 5;19(10):9958–70. doi: 10.1021/acsnano.4c16007 (PMC11924319; doi:10.1021/acsnano.4c16007)
Supplement: Supplementary file 1 — nn4c16007_si_001.pdf [file nn4c16007_si_001.pdf]

Supporting Information

Nanobody-targeted conditional antimicrobial therapeutics

Chayanon Ngambenjawong[1,2,3], Henry Ko[1,2], Tahoura Samad[1,2], Novalia Pishesha[1,4], Hidde L. Ploegh[5], Sangeeta N. Bhatia[1,2,6-9]*

[1]Koch Institute for Integrative Cancer Research, Massachusetts Institute of Technology, Cambridge, MA 02139, USA.

[2]Institute for Medical Engineering and Science, Massachusetts Institute of Technology, Cambridge, MA 02139, USA.

[3]School of Biomolecular Science and Engineering, Vidyasirimedhi Institute of Science and Technology (VISTEC), Rayong 21210, Thailand.

[4]Division of Immunology, Boston Children's Hospital, Harvard Medical School, Boston, MA 02115, USA.

[5]Program in Cellular and Molecular Medicine, Boston Children's Hospital, Harvard Medical School, Boston, MA 02115, USA.

[6]Howard Hughes Medical Institute, Cambridge, MA 02139, USA.

[7]Department of Electrical Engineering and Computer Science, Massachusetts Institute of Technology, Cambridge, MA 02139, USA.

[8]Department of Medicine, Brigham and Women's Hospital and Harvard Medical School, Boston MA 02115, USA.

[9]Broad Institute of Massachusetts Institute of Technology and Harvard, Cambridge, MA 02139, USA.

*Address correspondence to sbhatia@mit.edu.

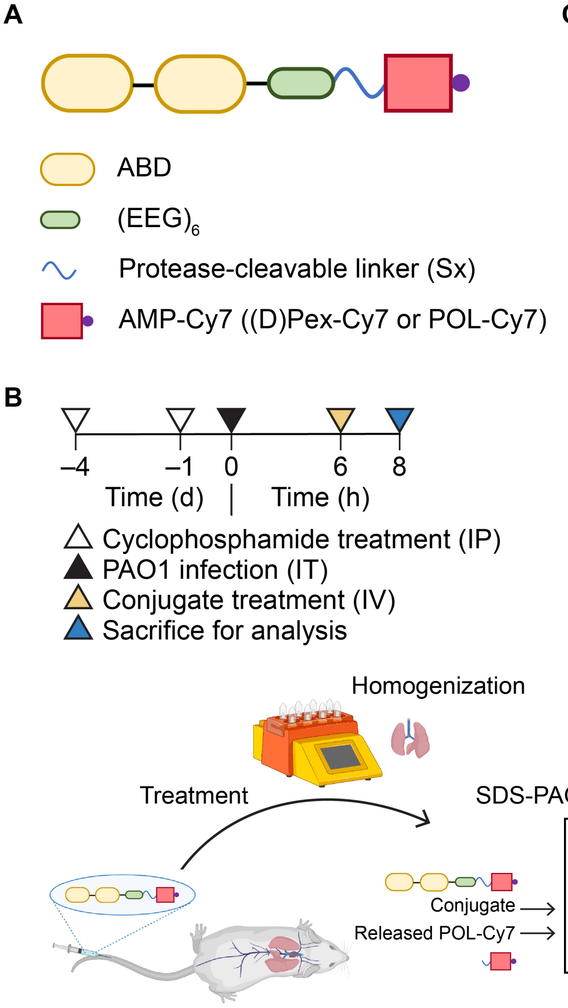

C

Round	Substrate	Sequence	Proteases known to cleave
1	S1	PLGLRSW	Thrombin, Cathepsins, MMPs
	S2	KPILFFRL	Cathepsin D/E
	S3	KAFRRSG	Cathepsins, Klk1
	S4	TTFYRRGA	Klk1
	S5	ARLYSRG	Klk1
	S6	TSVLMAAPQ	Napsin
	S7	VGPSQG	FAP
2	S5	ARLYSR	Klk1
	S8	VRFRST	Klk13
	S9	IQQRSL	Klks
	S10	RQSRIV	Klks
	S11	LAQAFRS	Klks, MMPs, ADAM10/17
	S12	TRFYSR	Klk1
3	S11	LAQAFRS	Klks, MMPs, ADAM10/17
	S13	LAQAVRS	ADAM10/17
	S14	LAQAFTS	ADAM17
	S15	LAAAVVS	ADAM17
	S16	KIEAVKS	ADAM10/17
	S17	PRAEALK	ADAM10/17

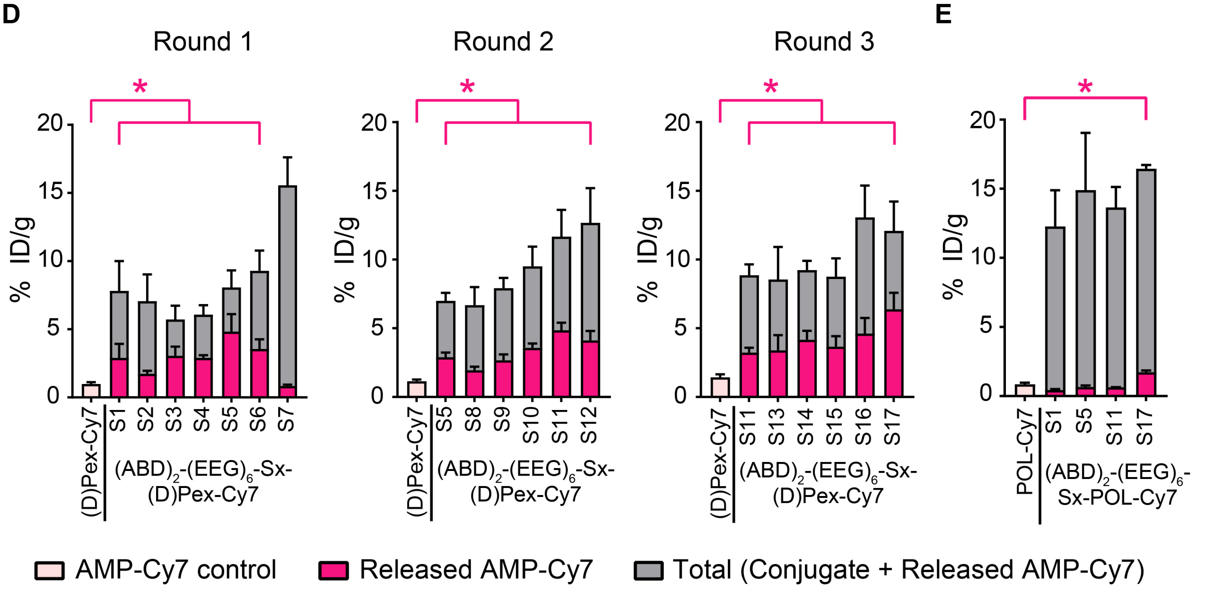

Figure S1. Optimization of cleavable linker improves conditional therapeutic activation.

(A) Design of ABD-AMP conjugates $(ABD)_2$-$(EEG)_6$-Sx-AMP-Cy7. Components: albumin-binding domain (ABD), anionic block ($(EEG)_6$), protease-cleavable linker (Sx), Cy7-labeled antimicrobial peptide (AMP-Cy7), model AMP-Cy7 ((D)Pex-Cy7 and POL-Cy7). (B) Experimental timeline and workflow for *in vivo* evaluation of biodistribution and activation of AMP-Cy7 conjugates. (C) List of cleavable linker substrates used in each round of screening. Quantification of total and activated fractions of (D) $(ABD)_2$-$(EEG)_6$-Sx-(D)Pex-Cy7 and (E) $(ABD)_2$-$(EEG)_6$-Sx-POL-Cy7 in PAO1-infected lungs presented as % injected dose (ID)/ gram (g). Panels D and E were plotted as mean ± SD and analyzed with One-way ANOVA with Tukey post hoc tests. (n = 3). Selected comparisons between AMP-Cy7 and released AMP-Cy7 from the conjugates were shown in pink. * denotes statistical significance ($P < 0.05$). Panel B was partly created with BioRender.com. For round 1, we started with linkers responsive to diverse proteases. The hit linker from the first round (S5) was known to be cleaved by kallikrein 1 (klk1), so we selected more linkers known to be cleaved by klk1 for evaluation in the second round. For round 2, the hit linker (S11) was known to be cleaved by both klk1 and ADAM10/17. Hence, we focused round 3 evaluation on ADAM10/17-responsive linkers and identified the S17 linker as the best hit.

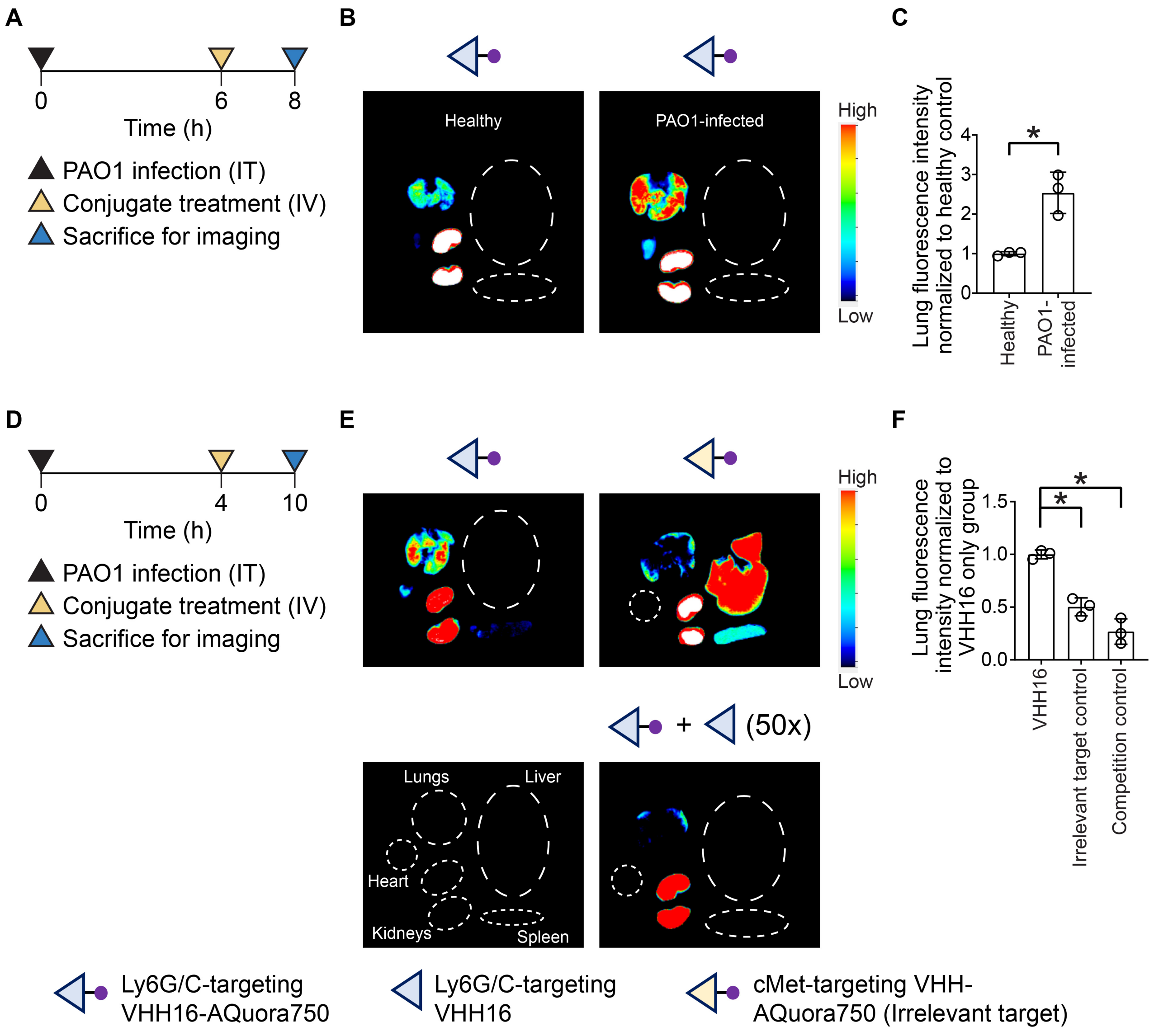

Figure S2. VHH16 accumulated in PAO1-infected lungs in an infection-dependent, VHH-specific manner. (A) Experimental timeline for *in vivo* biodistribution evaluation of VHH16-AQuora750 in healthy and PAO1-infected mice. (B) Representative *ex vivo* fluorescence images of VHH16-AQuora750 accumulation in different organs. (C) Quantification of VHH16-AQuora750 accumulation in healthy and PAO1-infected lungs reported as fluorescence intensity normalized to that of the healthy control. (D) Experimental timeline for *in vivo* biodistribution evaluation of VHH16-AQuora750, cMet-targeting VHH-AQuora750 (Irrelevant target control) and VHH16-AQuora750 + excess VHH16 (Competition control) in PAO1-infected mice. (E) Representative *ex vivo* fluorescence images of VHH-AQuora750 accumulation in different organs. (F) Quantification of VHH-AQuora750 accumulation in PAO1-infected lungs reported as fluorescence intensity normalized to that of the VHH16 only control. Panels C and F were plotted as mean ± SD and analyzed with One-way ANOVA with Tukey post hoc tests. (n = 3). * denotes statistical significance ($P < 0.05$).

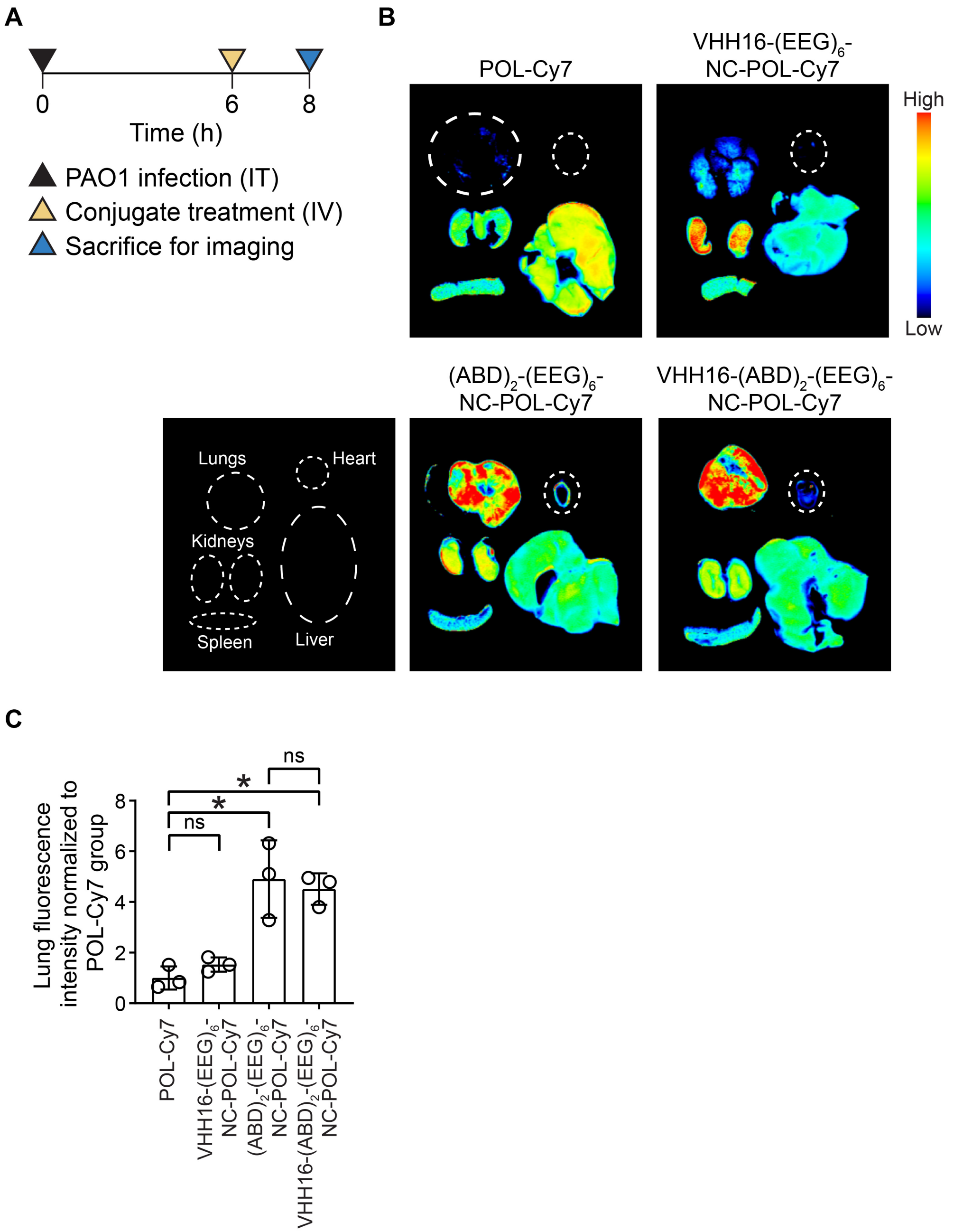

Figure S3. VHH16 did not increase accumulation of ABD conjugates in PAO1-infected lungs. (A) Experimental timeline for *in vivo* biodistribution evaluation of POL-Cy7 conjugates with non-cleavable linker (NC) in PAO1-infected mice. (B) Representative *ex vivo* fluorescence images of POL-Cy7 conjugate accumulation in different organs. (C) Quantification of POL-Cy7 conjugate accumulation in PAO1-infected lungs reported as fluorescence intensity normalized to the free POL-Cy7 control. Panels C was plotted as mean ± SD and analyzed with One-way ANOVA with Tukey post hoc tests. (n = 3). * denotes statistical significance ($P < 0.05$). NS denotes no statistical significance.

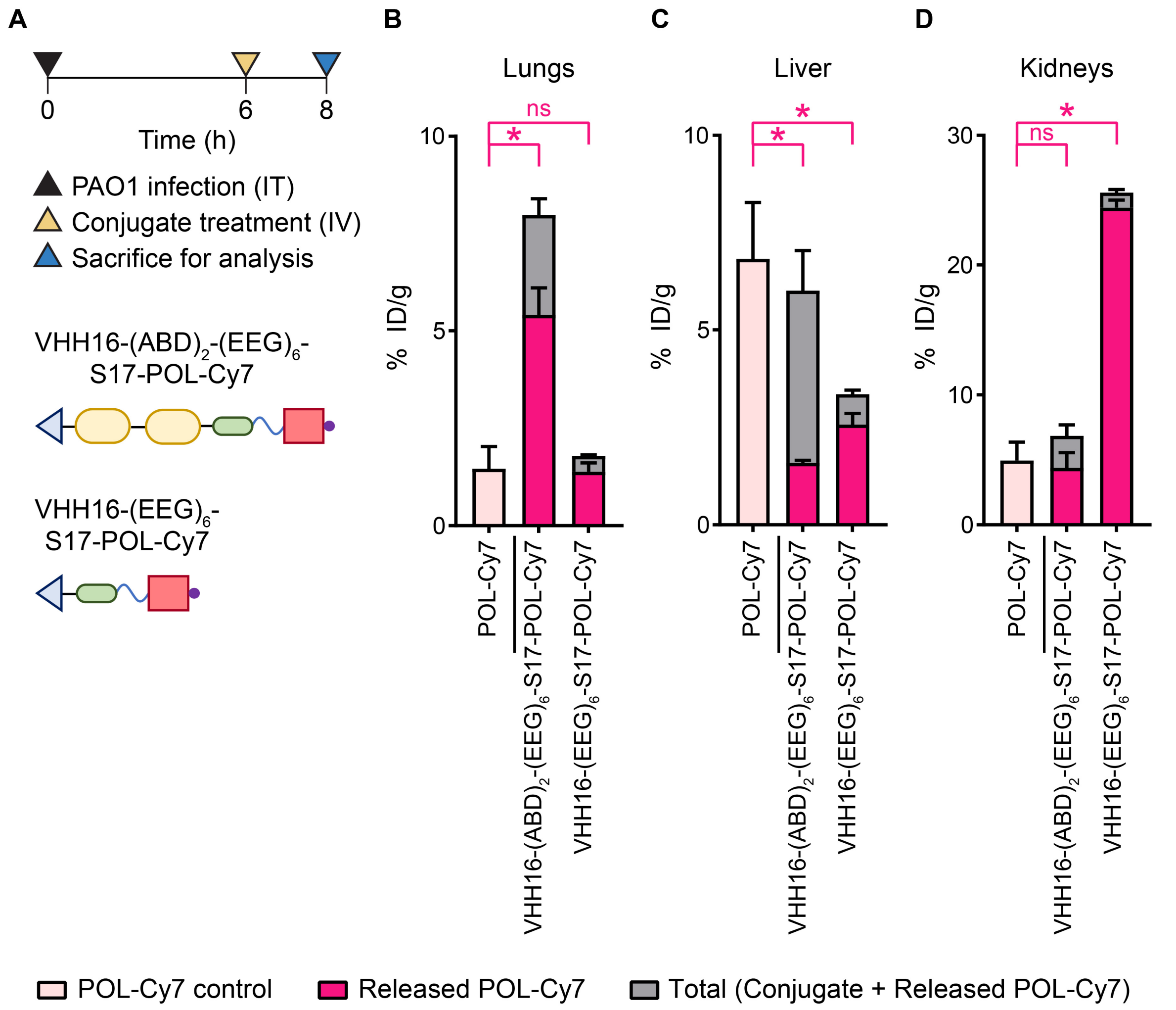

Figure S4. ABD is required to increase the amount of released POL-Cy7 of VHH16-targeted conjugate in PAO1-infected lungs.

(A) Experimental timeline for *in vivo* evaluation of biodistribution and activation of VHH16-targeted POL-Cy7 conjugates with and without ABD. Quantification of total and activated fractions of the POL-Cy7 conjugates in (B) PAO1-infected lungs, (C) liver, and (D) kidneys presented as % ID/g. Panels B-D were plotted as mean ± SD and analyzed with One-way ANOVA with Tukey post hoc tests. (n = 3). Comparisons between POL-Cy7 and released POL-Cy7 from the conjugates were shown in pink. * denotes statistical significance ($P < 0.05$).

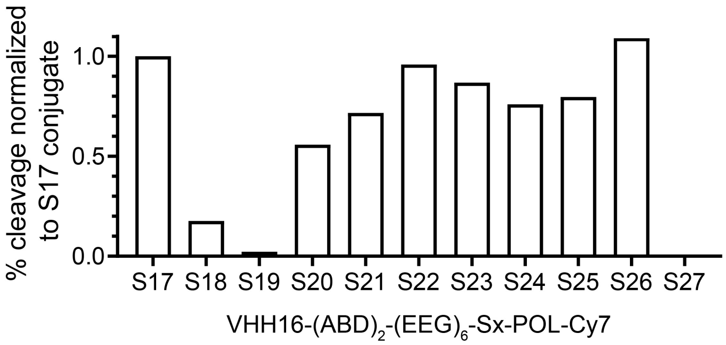

S17: PRAEALK (ADAMtide)
S18: PRAEAL-
S19: PRAEA--
S20: GRAEALK
S21: PRAAALK
S22: PRAEALT
S23: PRAEALV
S24: PRAEALA
S25: PRAEALS
S26: PRYEAYK (TENtide: ADAM10-specific)
S27: VFRMLSV (NE/PR3-sensitive, ADAM10-insensitive)

Figure S5. ***In vitro*** **ADAM10 cleavage assay identified tolerable mutations of the S17 linker.**
In vitro cleavages of VHH16-$(ABD)_2$-$(EEG)_6$-Sx-POL-Cy7 with different cleavable linkers by human ADAM10 were determined by SDS-PAGE analysis after incubation for 24 h and plotted as % cleavage normalized to that of the S17 conjugate.

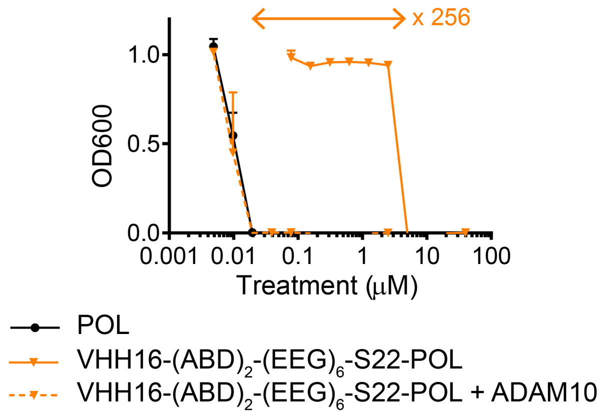

Figure S6. Activated VHH16-$(ABD)_2$-$(EEG)_6$-S22-POL has an equivalent antimicrobial potency as free POL. *In vitro* evaluation of antimicrobial activity masking of VHH16-$(ABD)_2$-$(EEG)_6$-S22-POL via microdilution assay on PAO1. Bacterial viabilities were measured based on OD600 absorbance normalized to the untreated control and plotted as mean ± SD. (n = 3).

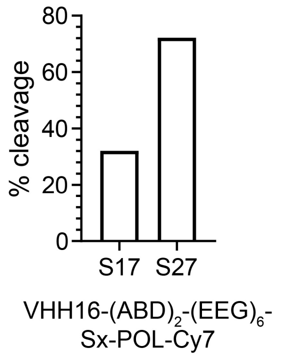

Figure S7. S17 and S27 POL-Cy7 conjugates can be activated to neutrophil elastase.
In vitro cleavages of VHH16-(ABD)$_2$-(EEG)$_6$-Sx-POL-Cy7 with different cleavable linkers by human neutrophil elastase (NE) were determined by SDS-PAGE analysis after incubation for 2 h and plotted as % cleavage.

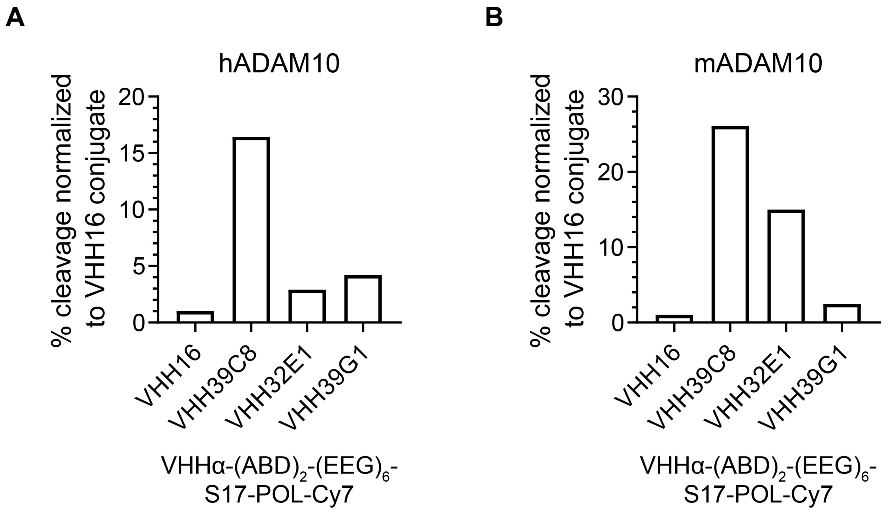

Figure S8. ADAM10-targeting VHHs enhance *in vitro* conjugate activation by ADAM10.
In vitro cleavages of VHHα-(ABD)$_2$-(EEG)$_6$-S17-POL-Cy7 with different ADAM10-targeting VHHs by human ADAM10 (left) and mouse ADAM10 (right) were determined by SDS-PAGE analysis after incubation for 2 h and plotted as % cleavage normalized to that of the Ly6G/C-targeted VHH16 control conjugate.

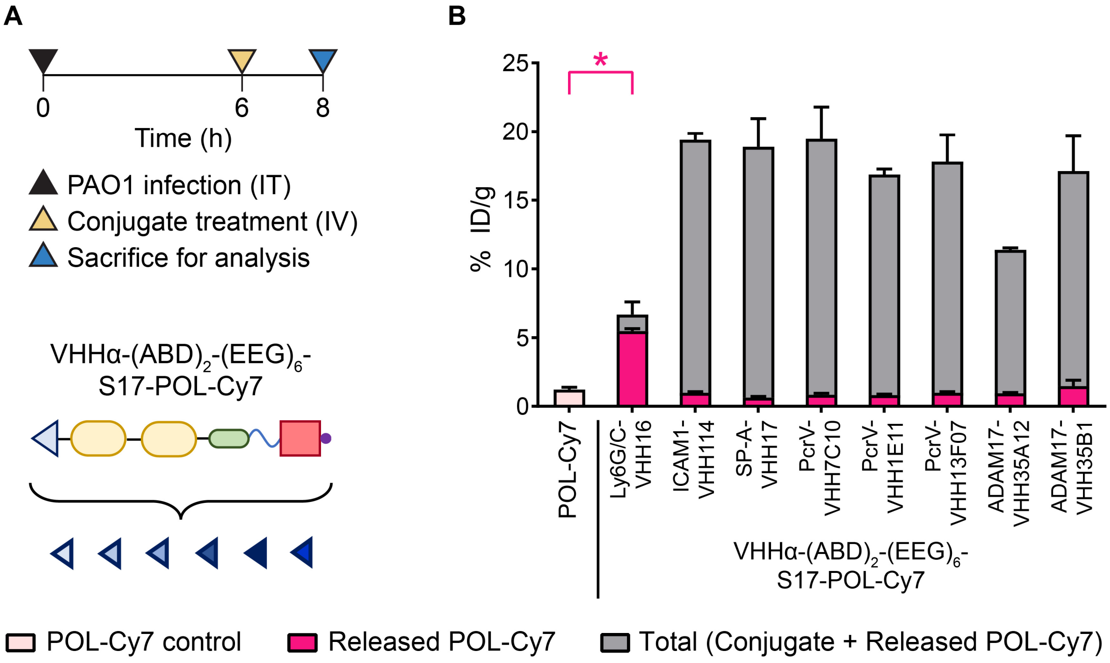

Figure S9. Expanded screening of VHHs with relevant targets for enhanced conjugate activation.
(A) Experimental timeline for *in vivo* evaluation of biodistribution and activation of VHHα-$(ABD)_2$-$(EEG)_6$-S17-POL-Cy7 with different targeting VHHs (VHHα). (B) Quantification of total and activated fractions of the POL-Cy7 conjugates in PAO1-infected lungs. Panel B was plotted as mean ± SD and analyzed with One-way ANOVA with Tukey post hoc tests. (n = 3). Only the amount of released POL-Cy7 from the VHH16 conjugate is statistically higher than that of the POL-Cy7 control indicated in pink asterisk. * denotes statistical significance ($P < 0.05$).

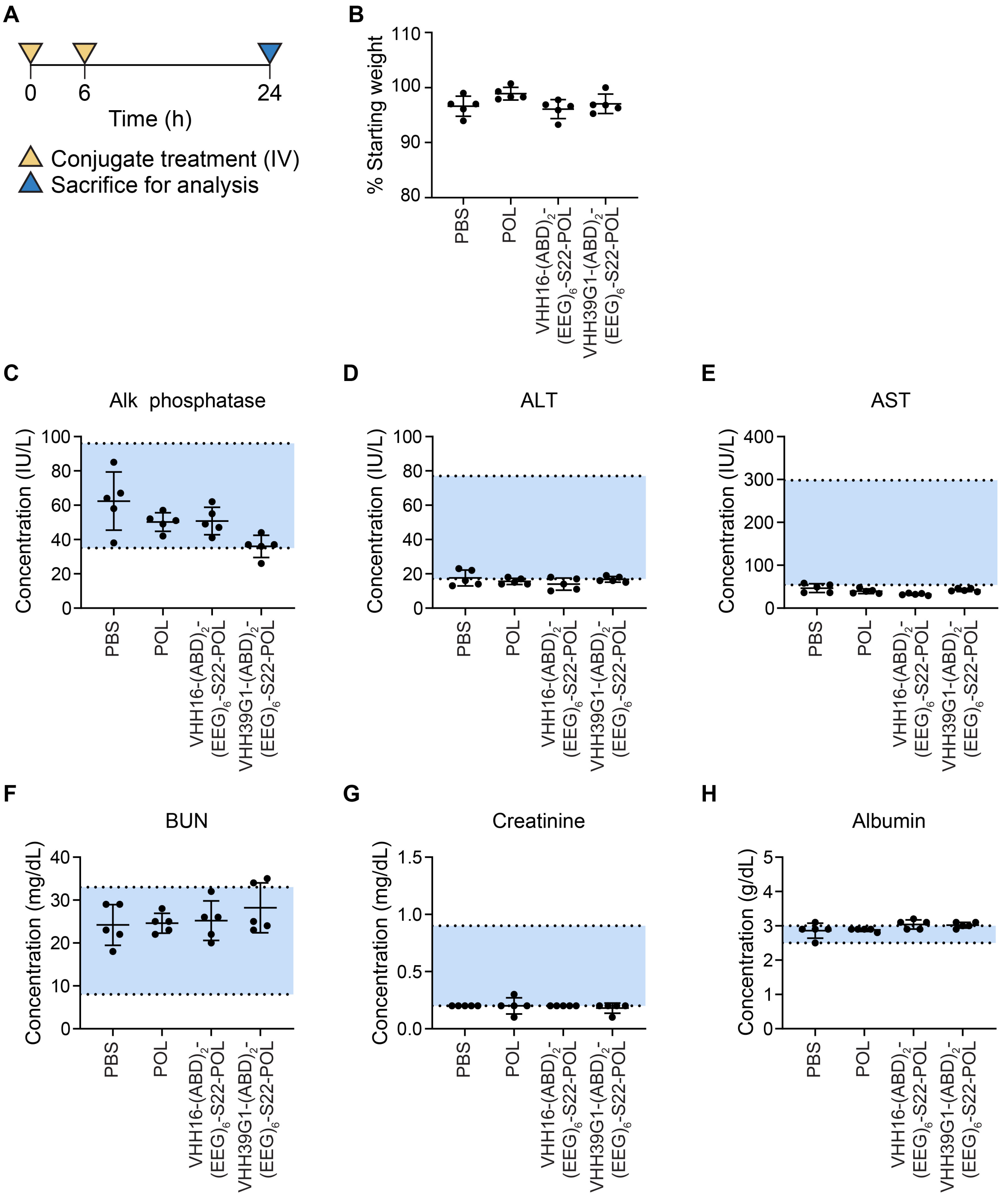

Figure S10. VHH-targeted POL conjugates exhibit good safety profiles.

(A) Experimental timeline for *in vivo* toxicity evaluation of VHH16-$(ABD)_2$-$(EEG)_6$-S22-POL and VHH39G1-$(ABD)_2$-$(EEG)_6$-S22-POL. Mice were treated intravenously with the conjugates at 5 mg/kg POL eq. twice (Total of 10 mg/kg POL eq. dose) and sacrificed at 24 h post first treatment to collect serums. (B) Percent body weight at the end point relative to the starting weight. Serum analysis of (C) Alkaline (Alk) phosphatase, (D) Alanine aminotransferase (ALT), (E) Aspartate aminotransferase (AST), (F) Blood urea nitrogen (BUN), (G) Creatinine, and (H) Albumin. Panels B-H was plotted as mean ± SD. (n = 5). Blue area indicates a normal reference range.

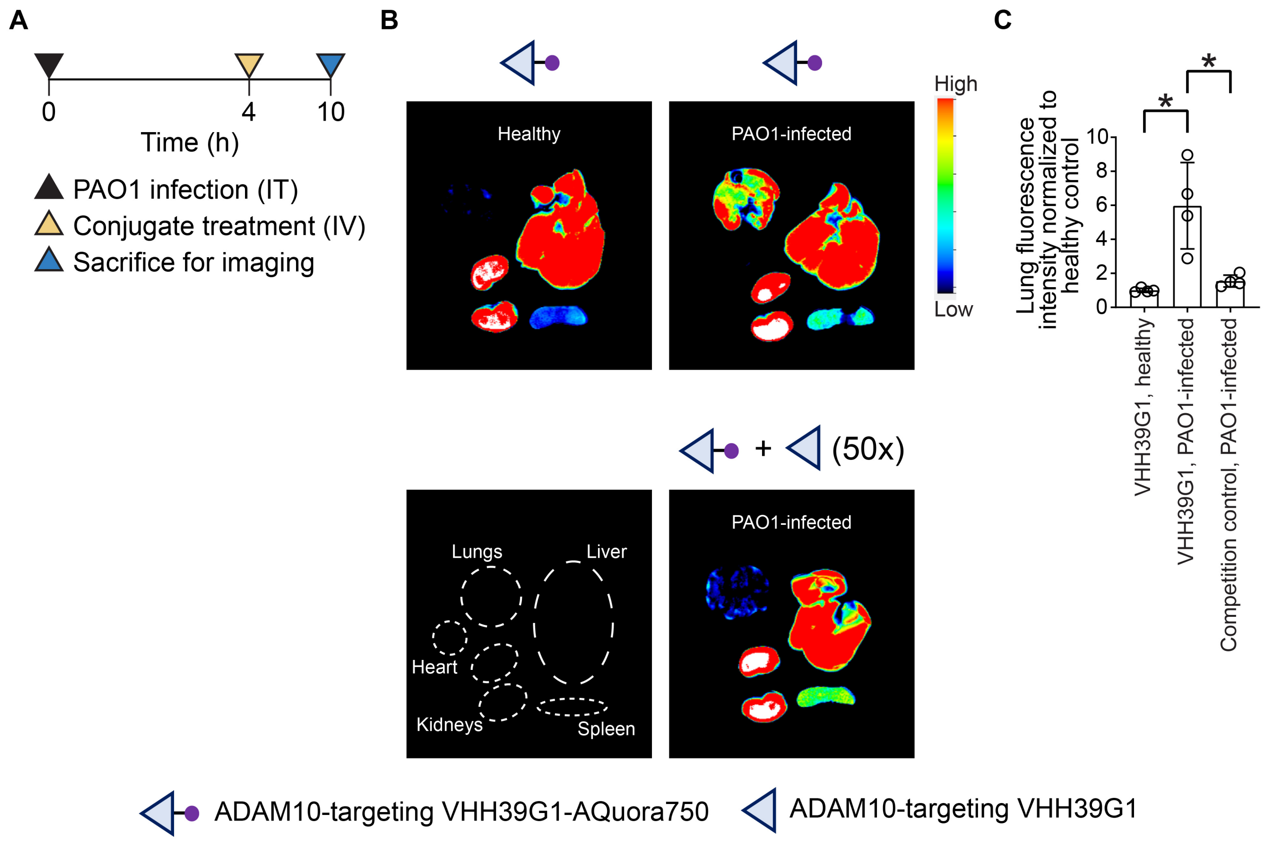

Figure S11. VHH39G1 accumulated in PAO1-infected lungs in an infection-dependent, VHH-specific manner.

(A) Experimental timeline for *in vivo* biodistribution evaluation of VHH39G1-AQuora750 in healthy and PAO1-infected mice without and with excess VHH39G1 (Competition control). (B) Representative *ex vivo* fluorescence images of VHH39G1-AQuora750 accumulation in different organs. (C) Quantification of VHH39G1-AQuora750 accumulation in healthy and PAO1-infected lungs without and with competition with excess VHH39G1 reported as fluorescence intensity normalized to that of the healthy control. Panel C was plotted as mean ± SD and analyzed with One-way ANOVA with Tukey post hoc tests. (n = 4). * denotes statistical significance ($P < 0.05$).

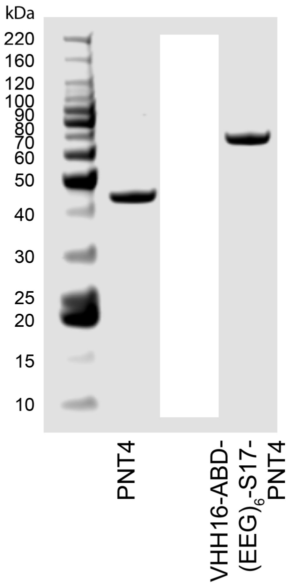

Figure S12. Recombinant VHH16-ABD-(EEG)$_6$-S17-PNT4 was readily expressed.
SDS-PAGE analysis of recombinantly expressed PNT4 and VHH16-ABD-(EEG)$_6$-S17-PNT4.

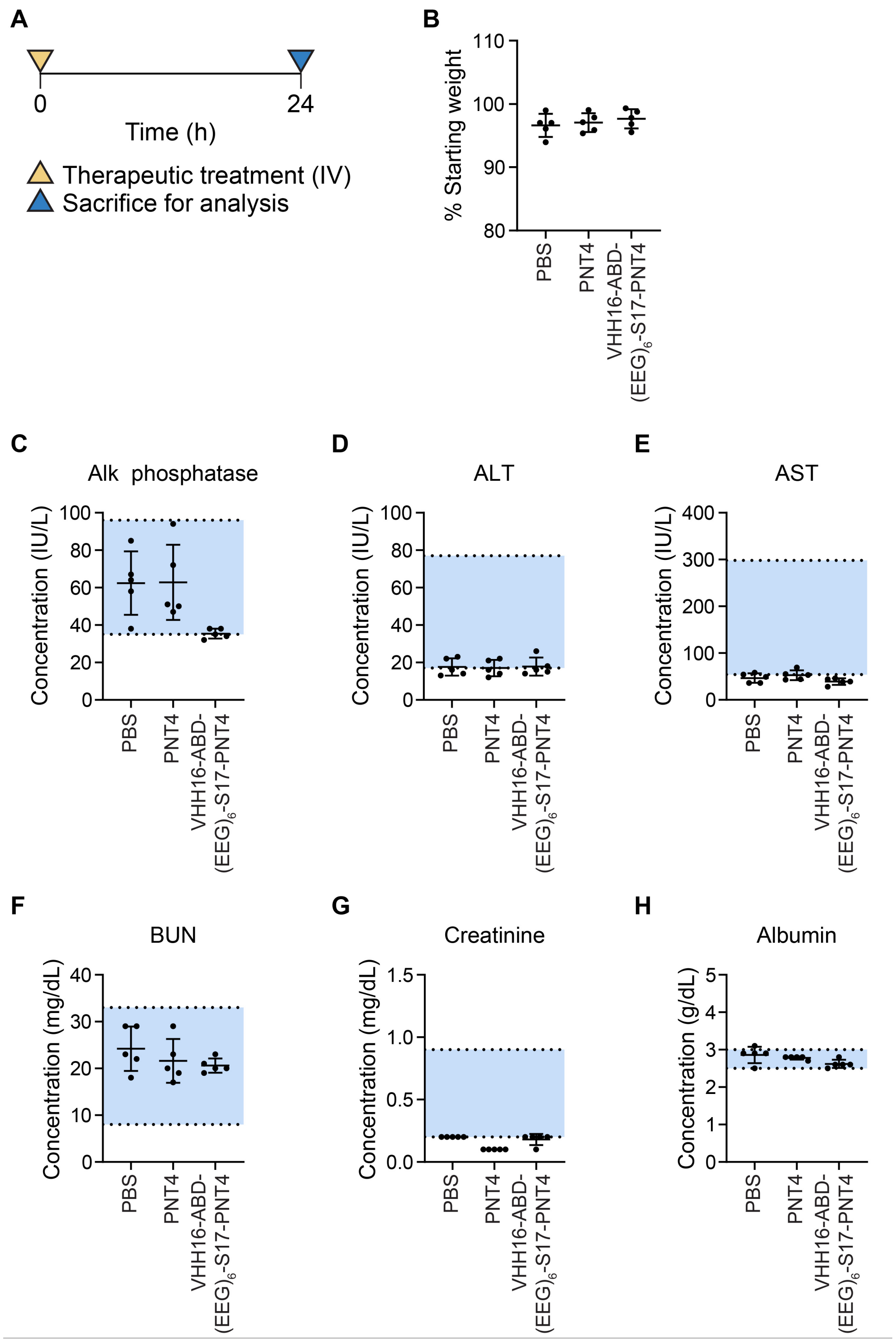

Figure S13. VHH-targeted conditional PNT4 exhibits a good safety profile.

(A) Experimental timeline for *in vivo* toxicity evaluation of VHH16-ABD-$(EEG)_6$-S17-PNT4. Mice were treated intravenously with VHH16-ABD-$(EEG)_6$-S17-PNT4 at 5 mg/kg PNT4 eq. and sacrificed at 24 h post treatment to collect serums. (B) Percent body weight at the end point relative to the starting weight. Serum analysis of (C) Alk phosphatase, (D) ALT, (E) AST, (F) BUN, (G) Creatinine, and (H) Albumin. Panels B-H was plotted as mean ± SD. (n = 5). Blue area indicates a normal reference range.

A

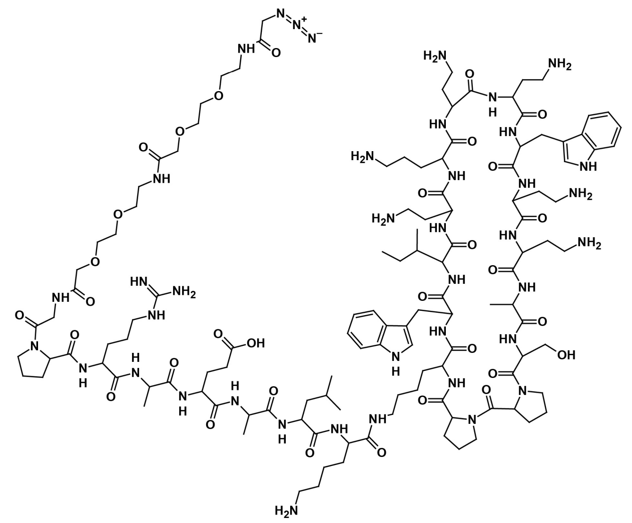

Cyclo-(K(Azidoacetyl-PEG2-PEG2-GPRAEALK-)-W-I-(Dab)-Orn)-(Dab)-(Dab)-W-(Dab)-(Dab)-A-S-p-P)

B

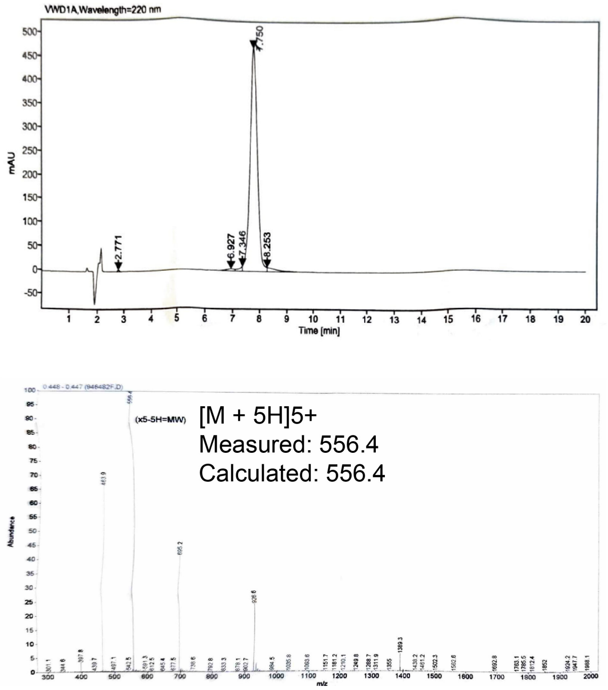

Figure S14. An example of POL peptide for conjugation.

(A) Structure and (B) liquid chromatography (Top) and mass spectrum (Bottom) of Azido-S17-POL.

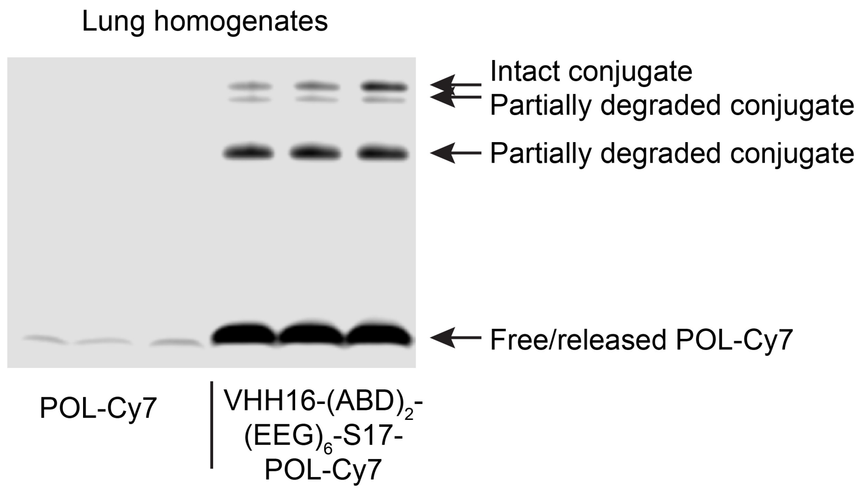

Figure S15. A representative SDS-PAGE image of lung homogenates from biodistribution study.

Table S1. List of therapeutic peptides and sequences.

Peptide	Sequence (N→C)
(D)Pex-Cy7-azide	Azidoacetyl–GiGkflkkakkfGkafvkilkk–K(Cy7)–CONH2
POL	Cyclo-(T-W-I-(Dab)-(Orn)-(Dab)-(Dab)-W-(Dab)-(Dab)-A-S-p-P)
POL-Cy7	Cyclo-(K(Cy7)-W-I-(Dab)-(Orn)-(Dab)-(Dab)-W-(Dab)-(Dab)-A-S-p-P)
POL-Cy7-azide	Cyclo-(K(N3)-W-I-(Dab)-(Orn)-(Dab)-(Dab)-W-(Dab)-(Dab)-K(Cy7)-S-p-P)
Azido-S17-POL	Cyclo-(K(Azidoacetyl-PEG2-PEG2-GPRAEALK-)-W-I-(Dab)-(Orn)-(Dab)-(Dab)-W-(Dab)-(Dab)-A-S-p-P)<br>Azidoacetyl-PEG2-PEG2-GPRAEALK was grafted from the Lys side chain (Highlighted in red)
Azido-S22-POL	Cyclo-(K(Azidoacetyl-PEG2-PEG2-GPRAEALT-)-W-I-(Dab)-(Orn)-(Dab)-(Dab)-W-(Dab)-(Dab)-A-S-p-P)<br>Azidoacetyl-PEG2-PEG2-GPRAEALT was grafted from the Lys side chain (Highlighted in red)
Azido-S28-POL	Cyclo-(T-W-I-(Dab)-(Orn(Azidoacetyl-G-Nle(O-Bzl)–Met(O)2–Oic–Abu-))-(Dab)-(Dab)-W-(Dab)-(Dab)-A-S-p-P)<br>Azidoacetyl-G-Nle(O-Bzl)–Met(O)2–Oic–Abu was grafted from the Orn side chain (Highlighted in red)

Note:
Cyclo indicates head-to-tail lactam cyclization.
Small letters denote D-amino acid.

Dab = 2,4-diaminobutyric acid
Orn = L-ornithine
Abu = L-2-aminobutyric acid
Nle(O-Bzl) = 6-benzyloxy-L-norleucine
Met(O)2 = L-methionine sulfone
Oic = Octahydroindole-2-carboxylic Acid
PEG2 = 2-(2-(2-Aminoethoxy)ethoxy)acetic acid

For the non-labeled therapeutic peptide conjugates, the cleavable linkers were synthesized in fusion to the therapeutic peptide to minimize the cleavage scar.

For the Cy7-labeled therapeutic peptide conjugates, the cleavable linkers were encoded in the recombinantly expressed fusion protein carrier VHHα-$(ABD)_2$-$(EEG)_6$-Sx-GC before DBCO-Mal functionalization and conjugation with AMP-Cy7-azide.

Table S2. List of VHH clones.

Target	Clone
Ly6G/C	VHH16, VHH21[1]
CD11b	VHH13[2]
ADAM10	VHH39C8, VHH32E1, VHH39G1[3]
ADAM17	VHH35A12, VHH35B1[3]
ICAM-1	VHH11-4[4]
SP-A	VHH17[5]
PcrV	VHH7C10, VHH1E11, 13F07[6]

References

(1) Bachran, C.; Schröder, M.; Conrad, L.; Cragnolini, J. J.; Tafesse, F. G.; Helming, L.; Ploegh, H. L.; Swee, L. K. The Activity of Myeloid Cell-Specific VHH Immunotoxins Is Target-, Epitope-, Subset- and Organ Dependent. *Sci. Rep.* **2017**, *7* (1), 17916. https://doi.org/10.1038/s41598-017-17948-0.

(2) Rashidian, M.; Keliher, E. J.; Bilate, A. M.; Duarte, J. N.; Wojtkiewicz, G. R.; Jacobsen, J. T.; Cragnolini, J.; Swee, L. K.; Victora, G. D.; Weissleder, R.; Ploegh, H. L. Noninvasive Imaging of Immune Responses. *Proc. Natl. Acad. Sci. U. S. A.* **2015**, *112* (19), 6146–6151. https://doi.org/10.1073/pnas.1502609112.

(3) Blanchetot, C.; Saunders, M. J. S.; De Haard, J. J. W. Amino Acid Sequences Directed against a Metalloproteinase from the ADAM Family and Polypeptides Comprising the Same for the Treatment of ADAM-Related Diseases and Disorders. U.S. Patent WO2008074840A2, October 13, 2015.

(4) Abulrob, A.; Arbabi-Ghahroudi, M.; Stanimirovic, D. Anti-ICAM-1 Single Domain Antibody and Uses Thereof. U.S. Patent WO2011134060A1, January 7, 2014.

(5) Wang, S.-M.; He, X.; Li, N.; Yu, F.; Hu, Y.; Wang, L.-S.; Zhang, P.; Du, Y.-K.; Du, S.-S.; Yin, Z.-F.; Wei, Y.-R.; Mulet, X.; Coia, G.; Weng, D.; He, J.-H.; Wu, M.; Li, H.-P. A Novel Nanobody Specific for Respiratory Surfactant Protein A Has Potential for Lung Targeting. *Int. J. Nanomedicine* **2015**, *10*, 2857–2869. https://doi.org/10.2147/IJN.S77268.

(6) De Tavernier, E.; Detalle, L.; Morizzo, E.; Roobrouck, A.; De Taeye, S.; Rieger, M.; Verhaeghe, T.; Correia, A.; Van Hegelsom, R.; Figueirido, R.; Noens, J.; Steffensen, S.; Stöhr, T.; Van de Velde, W.; Depla, E.; Dombrecht, B. High Throughput Combinatorial Formatting of PcrV Nanobodies for Efficient Potency Improvement. *J. Biol. Chem.* **2016**, *291* (29), 15243–15255. https://doi.org/10.1074/jbc.M115.684241.
